# Extending the information content of the MALDI analysis of biological fluids via multi-million shot analysis

**DOI:** 10.1371/journal.pone.0226012

**Published:** 2019-12-09

**Authors:** Maxim Tsypin, Senait Asmellash, Krista Meyer, Brandon Touchet, Heinrich Roder

**Affiliations:** Biodesix Inc., Boulder, Colorado, United States of America; Swiss Institute of Bioinformatics, SWITZERLAND

## Abstract

**Introduction:**

Reliable measurements of the protein content of biological fluids like serum or plasma can provide valuable input for the development of personalized medicine tests. Standard MALDI analysis typically only shows high abundance proteins, which limits its utility for test development. It also exhibits reproducibility issues with respect to quantitative measurements. In this paper we show how the sensitivity of MALDI profiling of intact proteins in unfractionated human serum can be substantially increased by exposing a sample to many more laser shots than are commonly used. Analytical reproducibility is also improved.

**Methods:**

To assess what is theoretically achievable we utilized spectra from the same samples obtained over many years and combined them to generate MALDI spectral averages of up to 100,000,000 shots for a single sample, and up to 8,000,000 shots for a set of 40 different serum samples. Spectral attributes, such as number of peaks and spectral noise of such averaged spectra were investigated together with analytical reproducibility as a function of the number of shots. We confirmed that results were similar on MALDI instruments from different manufacturers.

**Results:**

We observed an expected decrease of noise, roughly proportional to the square root of the number of shots, over the whole investigated range of the number of shots (5 orders of magnitude), resulting in an increase in the number of reliably detected peaks. The reproducibility of the amplitude of these peaks, measured by CV and concordance analysis also improves with very similar dependence on shot number, reaching median CVs below 2% for shot numbers > 4 million. Measures of analytical information content and association with biological processes increase with increasing number of shots.

**Conclusions:**

We demonstrate that substantially increasing the number of laser shots in a MALDI-TOF analysis leads to more informative and reliable data on the protein content of unfractionated serum. This approach has already been used in the development of clinical tests in oncology.

## Introduction

Plasma and serum proteomic profiling are valuable tools to assess the disease state of an organism [1–3], relating the relative abundance of circulating proteins to clinical data for diagnosis, prognosis, and treatment selection. We present a method for enhancing the sensitivity, reproducibility, and information content of measurements of the circulating proteome based on Matrix-Assisted Laser Desorption Ionization (MALDI) Time of Flight (TOF) mass spectrometry.

While there are many approaches attempting multiplexed measurements of protein abundance, for example, multiplexed immunoassays [4–8] and aptamer-based methods [9–13], most of these methodologies are targeted at a pre-defined set of known proteins assumed to be relevant for a particular disease state. In addition, circulating proteins are often post-translationally modified. Common modifications such as truncations, methylations, phosphorylations, splice isoforms, intrinsic oxidations etc., are not easily differentiable in classic antibody-based approaches [14–16]. These modifications can be important for the phenotypic state of disease [17], and disease specific effects may be missed when studies rely on measurements at the level of protein families. For example, in Wu et al [18] different modifications of serum amyloid A (SAA) were shown to be associated with gastric cancer when compared to gastritis and healthy patients. Differences in relative amounts of truncated forms of SAA have been observed in acute vs chronic inflammation [19] as well as in type 2 diabetes mellitus patients compared to non-diabetics [20].

In contrast to many other methods, mass spectrometry based proteomic profiling requires neither prior knowledge of disease mechanism nor a list of protein targets, and is capable of quantifying the relative abundance of hundreds of proteins simultaneously, including truncated and modified forms. A combination of mass spectral features (peaks) representing many different proteins/peptides can provide a robust way to discriminate between two clinical groups where individual features do not [21,22]. Successful application of multivariate data analysis and modern machine learning methods to mass spectrometry based proteomic data depends on the ability to simultaneously measure a large number of features in the mass spectra [23–29].

The use of proteome profiling of unfractionated serum with MALDI-TOF mass spectrometry provides several practical advantages. The required sample volume is very small (a few microliters of serum or plasma), enabling large scale experiments on archival sample sets where often only small volumes are available. Samples can be shipped either frozen or dried on paper cards, enabling the analysis of archival samples and providing an easy transport mechanism for potential clinical application. Data acquisition and analysis are high throughput. The same MALDI-TOF platform can be used for discovery, development and validation of tests, as well as for running the tests in the clinical setting.

The plasma and serum proteome is extremely complex, and its quantitative analysis presents unique challenges, mainly related to the wide range of protein concentrations, which can span more than 10 orders of magnitude [30–32]. The peak content of standard MALDI spectra of unfractionated serum is believed to be limited to about 150 peaks, associated with proteins (at masses above approximately 5 to 6 kDa) and peptides (at lower masses), including protein fragments and truncated forms, originating from highly abundant proteins [2]. An estimate of the range of protein abundances observable in standard MALDI-TOF experiments is about two to three orders of magnitude [33]. Quantitation of less abundant proteins is presumed difficult due to the limited dynamic range of MALDI-TOF [34], and is exacerbated by matrix-related chemical noise [35] and ion suppression effects [36–41]. Analytical reproducibility in MALDI protein profiling also remains a significant challenge [34, 42].

Fractionation techniques, such as multidimensional chromatographic separation coupled to mass spectrometry [43–52], could potentially improve the detection of low abundance proteins. However, such complicated multistep processes are time-consuming and hence not suitable for high-throughput applications; they require large sample volumes (from 10 μl [51] to 200–400 μl [48], typically 25–50 μl [46, 47, 49, 52]) and are difficult to reproduce, limiting the suitability for clinical applications. While approaches like multiple reaction monitoring (MRM) [53–58] can overcome some of the practical problems, these solutions require prior knowledge of useful proteins [59, 60].

In this work we study serum proteome profiling in the m/z range from 3 to 30 kDa using linear mode MALDI-TOF instruments. As we do not perform protein digestion, the proteins outside this mass range (i.e. heavier than 30 kDa) can only be observed via their naturally occurring fragments and truncated forms. Regarding the feasibility of proteome profiling using other types of mass spectrometers, linear MALDI-TOF remains a mainstream option. The m/z that we are studying are too high for a reflectron MALDI-TOF. Another promising possibility is Fourier transform ion cyclotron resonance mass spectrometry (MALDI-FTICR MS). These instruments demonstrate extremely high resolution, which would be very beneficial for profiling purposes. Historically, MALDI-FTICR instruments could only be used for relatively low m/z, such as up to 2500 Da [61] or up to 4000 Da [62]. However, relatively recently, using the state of the art 15-Tesla MALDI-FTICR instrument, the m/z range has been extended to 6500 Da [63], then to about 15 kDa [64, 65], and eventually to about 20 kDa [66]. It remains to be seen whether MALDI-FTICR becomes more widely used for proteome profiling. In this work we limit ourselves to improving the sensitivity, dynamic range and reproducibility of serum proteome profiling with MALDI-TOF MS, which remains highly relevant for discovery and validation of new biomarkers, as well as for clinical applications in personalized medicine where throughput is an important consideration [34]. One of our primary goals is to be able to acquire MALDI-TOF mass spectra that would provide a good starting point for further analysis with modern machine learning methods [23–29].

The problem of expanding the information content of MALDI-TOF proteomic profiling with respect to the accessible abundance range, e.g., number of detectable peaks, while retaining accuracy and reproducibility of quantitation, can be viewed as a problem of improving the signal-to-noise ratio (SNR) of peaks. This calls for reduction of noise in MALDI-TOF spectra, which can be achieved by averaging spectra from a very large number of laser shots.

Traditionally, MALDI-TOF applications using serum or plasma use around 2000 laser shots. Averaging tens of thousands of laser shots to improve signal-to-noise ratios has been done for MALDI-MS-MS fragmentation spectra [67–71]. Averaging 10 spectra, 500 laser shots each, to improve the accuracy of mass measurements of peptides, using reflectron MALDI-TOF MS, has been done in [72]. Summation of 20000 laser shots (reflectron MALDI-TOF, m/z range from 1000 to 5000 Da) was used in [73, 74] to quantify N-glycans in human serum. We applied the spectrum averaging approach to linear MALDI-TOF and found that the method can be extended to use much higher numbers of laser shots—up to 10^8^ shots.

In this paper, we describe the deep MALDI approach which enables acquisition of MALDI-TOF spectra with many more laser shots than conventionally used, by acquiring a large number of spectra from within and across sample spots and averaging them together. We show that this leads to reduction of noise and of the CVs of feature intensities, resulting in an increase of peak content, SNR, dynamic range, and quantitative reproducibility of MALDI-TOF spectra. These effects can also be observed in appropriate measures of spectral information content, and in association of spectral features with biological processes, computed using set enrichment techniques [75]. We present data from two different MALDI-TOF instruments: Bruker Ultraflextreme and SimulTOF100.

## Materials and methods

### Samples and sample preparation

The spectra used for this study were acquired over multiple years as a part of the standard quality control process at Biodesix. Spectra of unfractionated human serum samples were acquired on MALDI-TOF instruments in linear mode. Peaks in the spectra reflect peptides and proteins originally present in the sample. For each batch of experimental samples, four separate preparations of a reference control sample were spotted: two at the beginning, and two at the end of each MALDI sample plate, resulting, in total, in acquisition of 248,350 raw spectra of reference samples. We used two reference samples: one with Ultraflextreme (we denote this sample by RS1 in the remainder of the paper) and another with SimulTOF100 (denoted by RS2). Each reference sample was created by pooling equal volumes of serum obtained from five healthy individuals, purchased from ProMedDx LLC (Norton, MA, USA).

To evaluate the performance of the proposed acquisition methods on a data set obtained from a diverse set of samples, we utilized spectral acquisitions from our mass spectrometer qualification procedure. This procedure uses a sample set consisting of 40 serum samples purchased from Oncology Metrics (Fort Worth, TX, USA), which were derived from the blood of colorectal cancer and lung cancer patients. This set is called the machine qualification set (MQS) in the remainder of the paper.

To evaluate the biological implications of the presented approach we used a set of samples with sufficient volume to obtain protein expression measurements for a panel of 1305 known proteins, the SOMAscan (SomaLogic, Boulder, Co). 100 serum samples were purchased from the commercial biobanks Conversant Bio (Huntsville, AL) and Oncology Metrics (Fort Worth, TX). Samples were collected under ethics-approved protocols according to the requirements of Conversant Bio and Oncology Metrics. This set is called biological reference set (BR) in the remainder of the paper.

All samples used in this study have been approved for use in this study.

Sample preparation reagents acetonitrile (Burdick and Jackson), HPLC grade water (JT Baker), trifluoroacetic acid (EMD), and centrifugal filters were purchased from VWR International. Sinapinic acid was purchased from Sigma (St Louis, MO, USA) or Proteochem (Loves Park, IL, USA) and used without further purification. Serum cards and punches were purchased from Therapak (Claremont, CA, USA) and Acuderm (Ft Lauderdale, FL, USA), respectively, and Protein Calibration Standard I was purchased from Bruker Daltonics (Billerica, MA, USA).

### Instruments and instrument qualification

Two MALDI TOF mass spectrometers from different manufacturers were used for serum analysis in this study: Ultraflextreme (Bruker Daltonics, Bremen, Germany) and SimulTOF100 (SimulTOF Systems, Marlborough, MA, USA).

In order to obtain comparable spectra on different instruments and over extended periods of time, we have established a procedure to evaluate instrument performance. This is necessary as instrument performance will inevitably vary with normal wear and tear, repairs, and cleaning. Briefly, spectra are acquired from the machine qualification set and the reference control sample, and processed following a standardized sample preparation protocol. (Details on these procedures are provided in the S1 Appendix: Sample preparation and spectral acquisition). Feature values (integrated peak intensities) from spectra of each qualification and reference sample are compared to baseline acquisitions or “gold standard” spectra. Instrument parameters are tuned or adjusted until settings produce feature values concordant with the gold standard baseline acquisition.

### Spectral processing

#### Generation of averages

The raw data generated by the instrument is stored in the form of raw spectra, containing the sum of 800 laser shots each. In our experience, up to about 100 raw spectra can be acquired from each sample spot. Almost all spots allow acquisition of at least 50000 shots, before the sample is exhausted. To obtain average spectra for higher number of shots, we acquire raw spectra from multiple spots. This produces a pool of raw spectra which we align and use to obtain final average spectra. To generate averages without losing resolution, the raw spectra need to be aligned. A set of internal calibration points were selected that were detected in the majority of raw spectra using a SNR threshold of 3 for peak detection, and used to generate aligned spectra for averaging. Raw spectra that could not be properly aligned were excluded from further analysis. Average spectra were created by randomly selecting, without replacement, a fixed number of aligned raw spectra to achieve a predefined shot number. For example, to generate an 800,000 shot average, 1,000 raw spectra were included from the total pool of raw spectra acquired from multiple sample spots.

#### Spectral processing of averages

Preprocessing techniques were employed to allow comparison of averaged spectra, including background estimation and subtraction, alignment, and normalization.

Background was estimated using the convex hull method [76–78], and subtracted. Averaged spectra were re-aligned using peaks common to all spectra. Normalization was performed to adjust for overall intensity differences. We normalized spectra using the integrated intensity of background subtracted spectra over the union of three mass ranges: [6100, 7500], [8500, 10700], and [13300, 16400]. (All values in Da).

Each feature (typically containing a single peak) was defined by its left and right m/z boundary. Feature values are computed as the integrated intensity between the boundaries (sum of intensities of the mass spectral signal) for each feature and spectrum independently. Feature boundaries were designed to allow for variations in peak width and slight shifts in alignment. In this study, we predominantly focus on a set of features that are observable across all samples and acquisitions. This set contains 298 features listed in the S1 Appendix, unless otherwise stated.

#### Noise estimation

Noise in our mass spectra is defined as fluctuations around a mean value with a wavelength (much) smaller than the peak-width. For large numbers of laser shots the spectra become quite smooth, and we needed to use extra care to estimate these fluctuations. First, we isolate high-frequency noise, by computing the smoothed spectrum, using Savitzky-Golay smoothing [79] (window length = 29, polynomial order = 8), and subtracting the smoothed spectrum from the original spectrum. Then, to estimate noise at a given m/z, we consider all intensity values from data points within an m/z window of relative width 0.08 centered around this m/z value. For example, to estimate noise at 12 kDa, the m/z window is from 11520 to 12480 Da. We estimate the standard deviation of noise as the difference between the 50-th and the 25-th percentiles of this data, divided by 0.6745. This provides an estimate of the noise strength that (1) is robust to possible outliers in the data, and (2) in the special case of the normal distribution *N*(*μ*, *σ*^2^) reproduces its standard deviation *σ*. Indeed, for the normal distribution *N*(*μ*, *σ*^2^) the difference between the 50-th percentile *z*_0.5_ and the 25-th percentile *z*_0.25_ is

$$z_{0.5}-\;z_{0.25}=\;\sigma \sqrt{2}\;{\text{erf}}^{-1}\left(0.5\right),$$

where erf^−1^(*x*) is the inverse error function, erf^−1^(0.5) ≈ 0.4769362762 [80]. Thus

$$\sigma =\frac{z_{0.5}-\;z_{0.25}}{\sqrt{2}\;{\text{erf}}^{-1}\left(0.5\right)}\approx \frac{z_{0.5}-\;z_{0.25}}{0.6745}.$$

### Analytical information measure

We have developed a measure of the information content of a feature, designed to characterize its ability to differentiate between different samples. With this goal in mind, we consider the ratio of variability between samples (biological variability) to variability in repeated measurements of the same sample (technical variability). If this ratio is low (close to one), the measurement cannot distinguish between samples, and thus we cannot expect to be able to extract from it any clinically useful information. Consider repeated mass spectrometric measurements (“runs”) of a set of samples. We define the information content, *S*_*j*_, of a single feature, j, as follows. Using indices i: sample index (1 … number of samples), j: feature index (1 … number of features), k: run index (1 … number of runs), and denoting by f(i,j,k) the feature value for sample i, feature j, and run k:

$$\begin{array}{ll} S_{j} & =\;{\text{log}}_{2}\left(\frac{standard\;deviation\left(all\;samples,\;all\;runs\right)}{average\;over\;samples\left(standard\;deviation\;for\;a\;sample\;over\;runs\right)}\right)\\ & ={\text{log}}_{2}\left(\frac{standard\;deviation\left(f\left(:,\;j,\;:\right)\right)}{average\;over\;i\left(standard\;deviation\left(f\left(i,j,:\right)\right)\right)}\right). \end{array}$$

Here we use Matlab-inspired notation: f(:,j,:) is the collection of (number of samples)*(number of runs) values of feature j for (all samples, all runs), and f(i,j,:) is a collection of (number of runs) values of feature j for all runs of sample i. The total information content for a mass spectrum is then just the sum of *S*_*j*_ over all features.

### Association of peaks with biological processes

The strength of association of mass spectral features with biological processes was estimated by applying the commonly used bioinformatics tool, gene set enrichment analysis (GSEA) [75], to protein expression. The set enrichment approach determines the association of a measured quantity (in this case a mass spectral feature value) with a particular biological process by looking for a consistent pattern of associations with the quantity in question across a set of proteins (or genes) known to be related to that biological process. Hence, to be able to associate individual mass spectral features with biological processes, it is necessary to have matched protein expression data and mass spectral data for a reference sample set. Relative protein abundance measurements for a panel of 1305 proteins were obtained for the BR set using the aptamer-based 1.3k SOMAscan assay (SomaLogic, Boulder, CO). Mass spectral data from the same samples were also collected as described in “Materials and methods”, and mass spectral feature values determined for each sample for a predefined set of 298 features (See “Spectral processing of averages”).

Protein sets for various biological processes of interest were defined as follows. The GeneOntology database, GO, (Gene Ontology Consortium) [81,82] was queried using AmiGO [83, 84] and EMBL-EBI QuickGO [85] web applications to perform ontology searches and create lists of gene products associated with biological processes of interest. Many processes are interrelated; for example, activation of the complement system and acute phase response are important parts of innate immunity, and some elements of these lists inevitably overlap. This redundancy reflects the common aspects of related pathways. Typically, we selected relationships to the annotated terms that included “is a”, “part of”, “occurs in”, and “regulates”; however, when this choice seemed too broad, we used narrower relationships. Evidence was filtered to allow for all types of manually reviewed annotations, but to exclude “electronic” annotations (not manually reviewed; evidence code “IEA” [86]). The intersection of the set of proteins found to be associated with a GO biological function of interest and the proteins measured in the SOMAscan panel yielded the protein set for this particular biological function. A table of the biological functions considered and their associated protein sets is provided in S1 Appendix.

The protein set enrichment analysis (PSEA) approach [87] first determined the univariate correlation between the values of a mass spectral feature and each of the 1305 proteins measured by the SOMAscan panel within the BR set. These univariate associations were assessed using the Spearman correlation coefficient. From these correlations, an enrichment score was generated, which assessed the relative consistency of the univariate correlations for the proteins contained in the protein set for the biological process in question compared with that for proteins measured but not contained in the relevant protein set. The enrichment score was defined as in [88] as this approach provides increased power for the identification of associations compared with the standard GSEA method [75]. P-values of association between each mass spectral feature and the biological processes were obtained by comparing the enrichment score with the null distribution generated by random permutation of the features values across the sample set. This approach followed the standard GSEA method described in [75]. False discovery rates for this multiple testing problem were estimated using the method of Benjamini-Hochberg [89].

## Results

The numbers of raw spectra (800 laser shots each) available for averaging and further analysis are summarized in Table 1. As described in “Materials and methods”, we randomly selected fixed numbers of these raw spectra to generate averages for fixed numbers of laser shots up to 100 million (for RS2 on the SimulToF instrument).

**Table 1:** The number of raw spectra in each raw spectra pool for the different samples and instruments.

Sample/instrument	Number of raw spectra in the raw spectra pool	Total number of laser shots
RS1 Ultraflextreme	28,283	22.6×10^6^
RS2 SimulTOF100	220,067	176×10^6^
MQS SimulTOF100	794,994 (overall); 16,511…20,706 (per sample)	636×10^6^ (overall); (13.2…16.5)×10^6^(per sample)
BR SimulTOF100	189,595 (overall); 1,686…2,012 (per sample)	152×10^6^ (overall); (1.35…1.61)×10^6^ (per sample)

**Fig 1:**
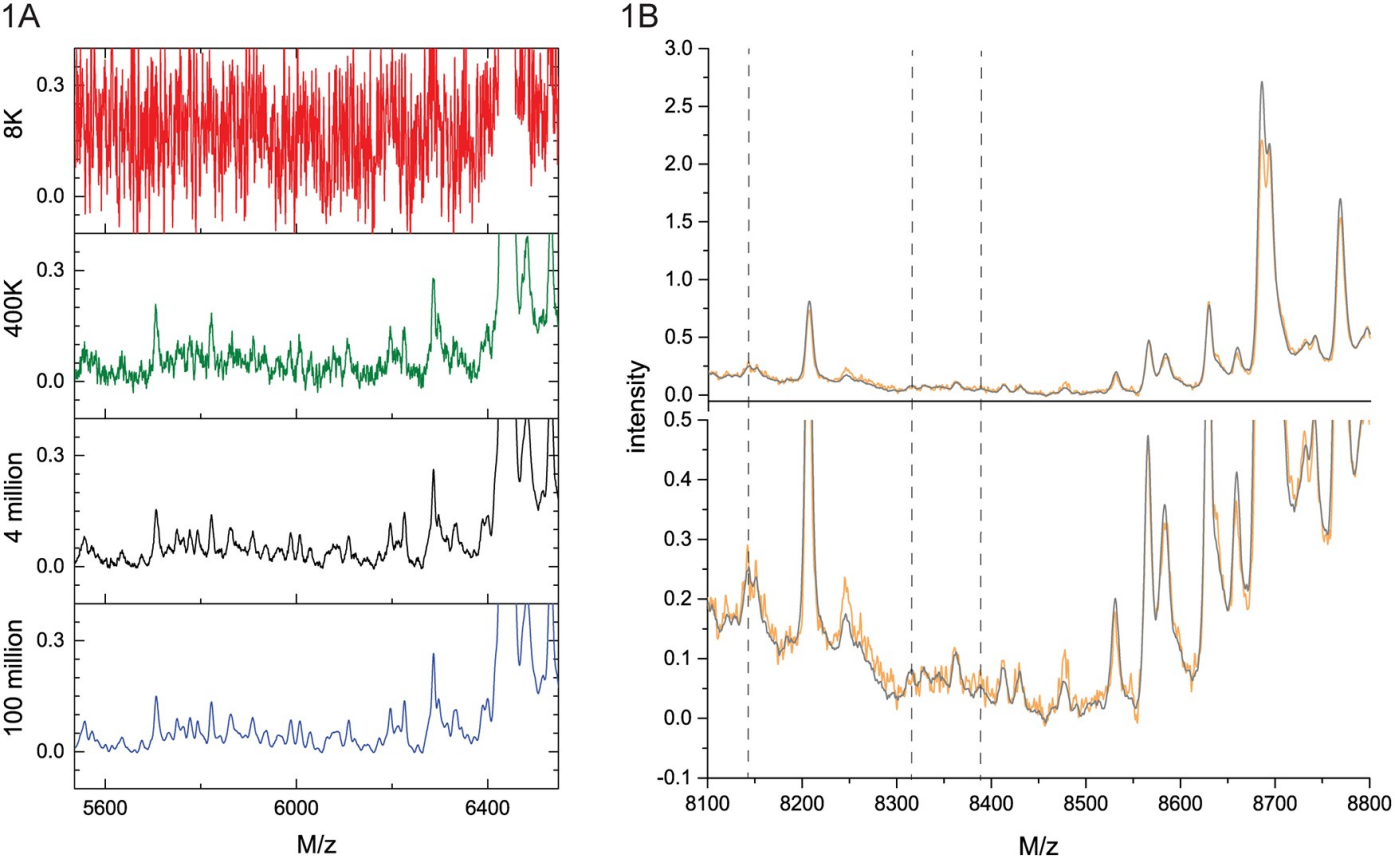
Dependence of average spectrum on the number of laser shots. A) Processed spectra for the reference sample RS2 acquired on the SimulTOF100 are shown in the mass range from 5.53 kDa to 6.55 kDa for 8K, 400K, 4 million, and 100 million laser shots. B) Comparison of average spectra for the reference sample RS2 acquired on the SimulTOF100 in the m/z-range 8.1 to 8.8 kDa. Orange: 0.4 million laser shots, Black: 8 million laser shots. *Top panel*: The whole intensity range; *Bottom panel*: Enlarged to show low-intensity peaks. The dashed vertical lines indicate the m/z position of small peaks discussed in the text.

The dependence of the averaged spectra on the number of shots for the RS2 acquisition acquired on the SimulTOF100 is shown in Fig 1A. While there are no distinguishable peaks in the 8000 shot spectrum (in the selected mass range), small peaks emerge from the noise as the number of shots is increased; the peaks become better defined and differentiable, and the noise decreases. The last point is better illustrated by comparing averaged spectra including different numbers of shots and zooming into the y-axis as shown in Fig 1B. As the number of shots increases from 400 thousand to 8 million shots, the noise is greatly reduced, enabling the detection of small peaks (e.g. at 8320 and 8380 Da) and the differentiation of close peaks (e.g. around 8140 Da). It is necessary to zoom into the intensity axis to see the small peaks due to the large range of protein abundances in human serum [30–32] visible in the deep MALDI averages.

Assuming that abundance and peak intensity are proportional, and neglecting possible ion-suppression [36–41], our estimate of the observable dynamic range in our acquisition is about 4 orders of magnitude, as measured by the ratio of the largest observable peak to the smallest observable peak. (For comparison, at 8000 laser shots the observable dynamic range is about 2 orders of magnitude). This shows that it is possible to directly measure low abundance proteins in the presence of high abundance proteins with MALDI-TOF without fractionation, as long as the respective peaks are well resolved in m/z.

### Dependence of SNR and number of observable peaks on shot number

To further investigate the characteristics of the spectra as a function of number of shots, we analyzed how noise varies with increasing number of laser shots. According to the law of large numbers and assuming ideal experimental conditions, the noise should decrease as the square root of the number of laser shots. Indeed, consider the average spectrum $\bar{y}\left(x\right)$ obtained by averaging *n* spectra *y*_*i*_(*x*):

$$\bar{y}\left(x\right)=\;\frac{1}{n}\overset{n}{\underset{i=1}{\sum }}y_{i}\left(x\right)\;,$$

where *x* = *m*/*z*, and *i* = 1 …*n* is the index of the spectrum. Individual spectra *y*_*i*_(*x*) contain signal *s*(*x*) and noise *r*_*i*_(*x*):

$$y_{i}\left(x\right)=s\left(x\right)+r_{i}\left(x\right).$$

The signal *s*(*x*) is the same for all spectra, thus

$$\bar{y}\left(x\right)=s\left(x\right)+\bar{r}\left(x\right),$$

where

$$\bar{r}\left(x\right)=\;\frac{1}{n}\overset{n}{\underset{i=1}{\sum }}r_{i}\left(x\right).$$

At any given *x*, each of *r*_*i*_ is independently drawn from the same probability distribution, characterized by expected value *E*[*r*_*i*_] = 0 and variance Var(*r*_*i*_) = *σ*^2^. Thus

$$\text{Var}\left(\bar{r}\right)=\;\frac{1}{n}\text{Var}\left(r_{i}\right),$$

and its standard deviation

$$\sigma \left(\bar{r}\right)=\frac{1}{\sqrt{n}}\sigma \left(r_{i}\right).$$

This does not require the distribution of *r*_*i*_ to be Gaussian; however, due to the central limit theorem, for large *n* we expect the distribution of $\overset{-}{r}$ to be approximately Gaussian.

**Fig 2:**
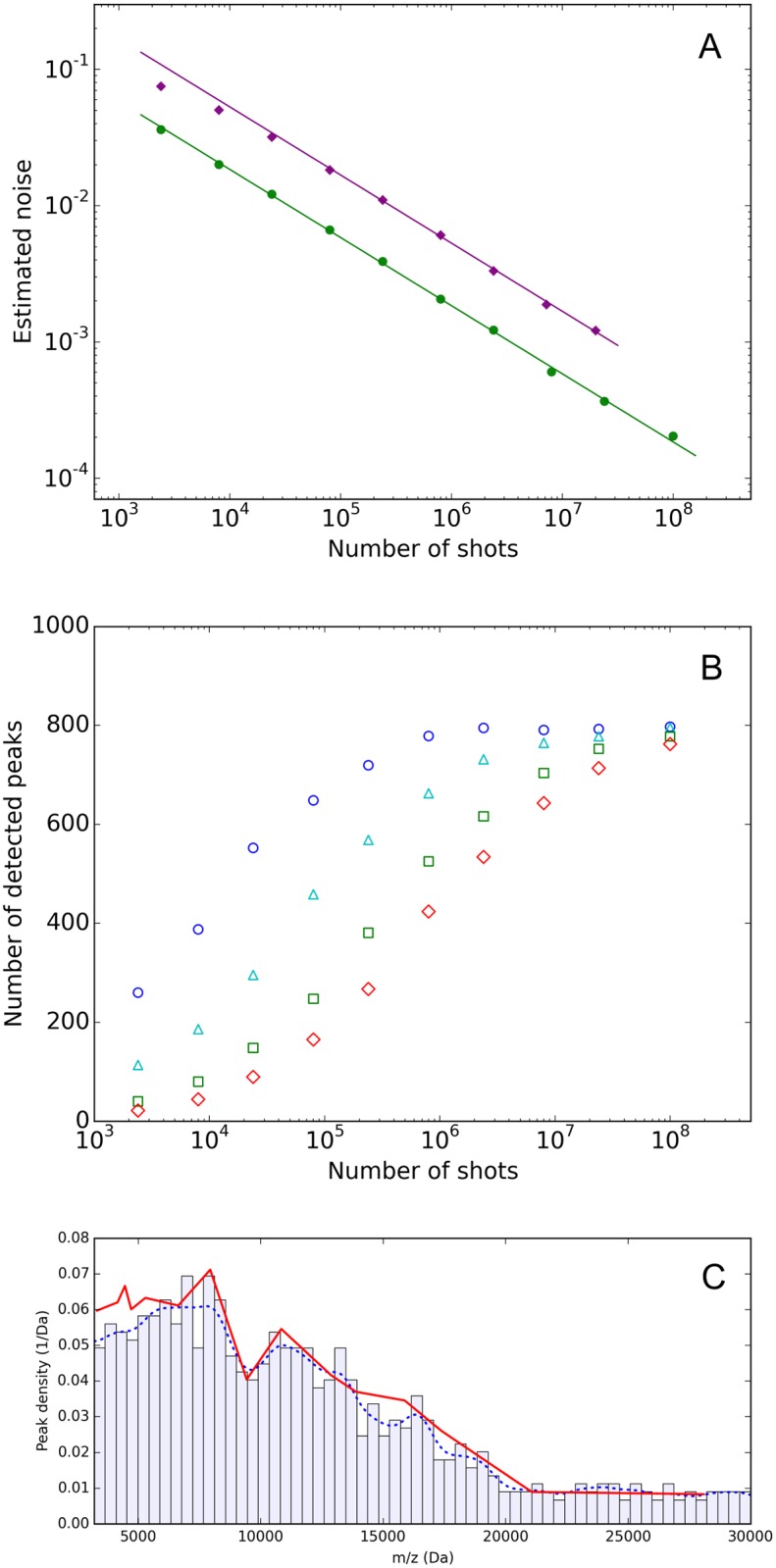
Noise level, number of detected peaks and peak density. A) Noise level in the spectra, as a function of the number of shots. Noise is estimated as described in “Materials and methods” and shown for RS1 obtained on the Bruker Ultraflextreme (purple) and for RS2 on the SimulTOF100 (green). The lines show the expected slope of -1/2 and are shifted by instrument dependent offsets. B) Number of detected peaks for a fixed SNR cutoff, as a function of the number of laser shots for RS2 on the SimulTOF (SNR = 10 (dark blue circles), 20 (light blue triangles), 40 (green squares), 60 (red diamonds)). C) Density of detected peaks at a SNR cut-off of 10 obtained by counting the number of peaks in equal-width m/z bins (the m/z range from 3200 to 30000 Da was divided into 60 bins), for the 100 million shot spectrum as a function of m/z. The dotted blue line is a smoothed version of this density, and the red line is proportional to the inverse peak width estimated from several most prominent peaks in the spectrum.

In Fig 2A, we show the estimated noise (see “Materials and methods”) as a function of the number of shots for RS1 acquired on the Bruker Ultraflextreme and RS2 on the SimulTOF100. For acquisitions on either instrument, the noise decreases over the whole accessible range of numbers of shots with the expected inverse square root behavior, indicating that increasing the number of laser shots and using the described averaging procedure efficiently reduces the amount of noise present in the average spectra, independent of the instrument.

We are interested in measuring as many peaks as possible with reasonable SNR cutoffs. In Fig 2B, we show the increase in the number of observable peaks as a function of laser shots for four different SNR cut-offs for RS2 acquired on the SimulTOF100. As expected, the number of observable peaks increases with increasing number of shots, but then surprisingly reaches a plateau at about 800 peaks. As the noise continues to decrease (see Fig 2A), this effect is at first glance surprising. We believe that the limit on the number of observable peaks is related to the finite resolution of the instrument, and that we are observing the effect of “peak crowding”. The masses of observable proteins are not uniformly distributed across the m/z axis and there are regions where there are more peaks that are too close together to be resolved by the instrument. Hence, we would not be able to distinguish peaks in these areas even if we had optimal sensitivity, and in our high-number-of-shots approach, the number of peaks as a function of shot number is primarily limited by the resolution of the instrument. This explanation is illustrated in Fig 2C which shows the density of peaks and compares this density with the estimated inverse peak width. Over the whole m/z range from 3 to 30 kDa the density of peaks appears to be proportional to the inverse of the peak width. This supports the idea that the number of observable peaks is limited by instrument resolution, rather than by its sensitivity. Of course, the underlying distribution of peaks depends on how many proteins are actually present in a sample in a given m/z interval. One would need to repeat these experiments using instruments with higher resolution to answer this question more definitively. Artificially reducing resolution, by smoothing the spectra using a moving average with window width of 41 points (in S1 Fig we compare such a smoothed spectrum with the original), we observe that the plateau in Fig 2B is reduced from around 798 peaks to 442 peaks, indicating that the number of observable peaks in MALDI serum spectra is limited more by resolution effects than sensitivity, if one utilizes many laser shots. Note that this effect is a manifestation of a high complexity of the sample (serum contains thousands of proteins or protein isoforms), whereas samples with lower complexity, e.g. spiked proteins in water, are not expected to be affected by peak crowding.

### Analytical reproducibility of peak intensity as a function of laser shots

For clinical applications it is important to have good analytical performance of the measurement process. Having demonstrated that substantially increasing the number of shots leads to a reduction in noise and an increase in the number of observable peaks, we now show how reproducibility improves with increasing number of shots. We needed to perform this experiment using a diverse set of samples to ensure that we were not confounded by peculiarities of a single sample. To demonstrate the improvement of analytical reproducibility with increasing shot number, we created two sets of averages for the 40 samples in MQS ranging from 2400 shots to 8 million shots (limited by the available number of raw spectra). We examined the reproducibility of the 298 feature values by comparing the two sets in concordance analyses.

**Fig 3:**
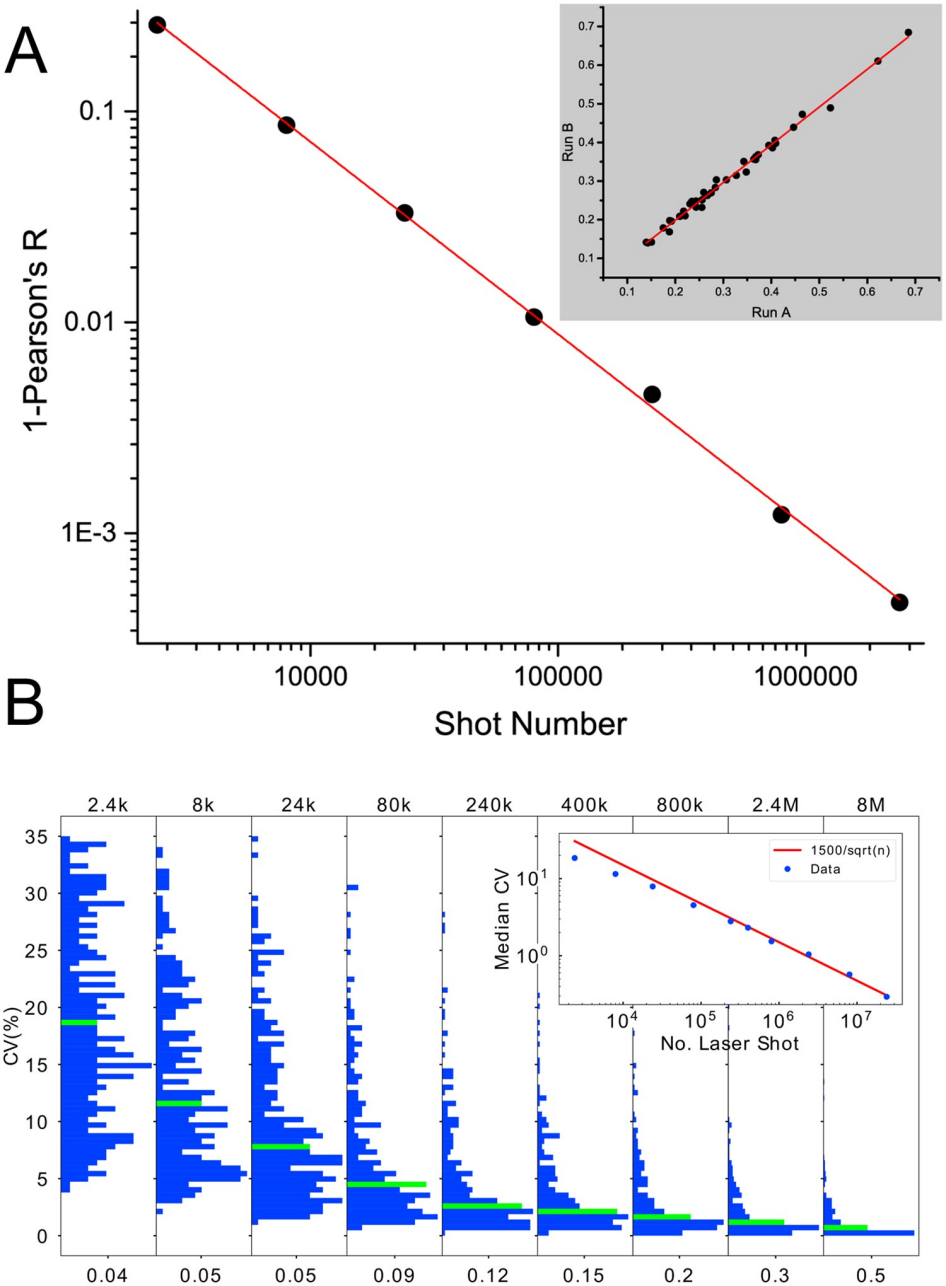
Reproducibility of feature values. A) The median of (1-Pearson’s R) over all peaks, as a function of the number of shots for the MQS acquired on the SimulTOF100. The inset shows an example of the concordance analysis for the peak at 10236 Da for 240,000 shots. In the inset the horizontal axis relates to feature values from the set 1 of the MQS averages (Run A), and the vertical axis to feature values of MQS set 2 averages (Run B). B) The distributions of CVs of all features (peaks) for increasing numbers of laser shots of RS2. For each number of shots the median CV is highlighted. The horizontal axis for each panel is the relative frequency of values of CVs. The histograms are normalized as probability densities (area under the histogram is equal to 1). For each histogram, the value on the horizontal axis is a half of the maximum value of the histogram. The inset shows the median CV as a function of the number of shots with the red line indicating a behavior proportional to the inverse square root of the number of shots.

We use linear regression analysis as a measure of concordance (perfect concordance would result in a slope of 1). In Fig 3A, we report the results of the concordance analysis showing the median of (1—Pearson’s R) from fits to a straight line in the feature concordance as a function of the number of laser shots. We see that as the number of shots increases, the median Pearson’s R becomes closer to one indicating that reproducibility as measured by feature concordance improves.

To obtain an additional measure for the analytical reproducibility as a function of number of shots, we also estimated the CVs of the 298 features using 20 replicate averages at different numbers of shots for the RS2. In Fig 3B we show the CV distribution of the features as a function of the number of shots. As the number of shots increases, both the range and the median of the distribution decrease systematically (see also Table 2), indicating that the reproducibility of mass spectral features improves with the number of laser shots. The median CV decreases as a power law in the number of shots with exponent 0.5, as expected.

**Table 2:** Median CVs and ranges for the distributions of Fig 3B as a function of number of shots.

Number of shots	Median CV	25th Quartile	75th Quartile
2,400	18.50	11.90	27.64
8,000	11.48	6.85	19.44
24,000	7.91	4.60	14.25
80,000	4.53	2.62	9.42
240,000	2.80	1.61	6.10
400,000	2.31	1.37	4.97
800,000	1.54	0.92	3.46
2,400,000	1.04	0.60	2.47
8,000,000	0.57	0.29	1.50

### Information content of mass spectra as a function of laser shots

One primary application of MALDI proteome profiling is the development of tests based on the measurements of the abundance of circulating proteins, without requiring prior selection of specific target proteins. Successful development of such tests depends on the richness of the information content of the underlying data. Here we attempt to assess the dependence of the information content of spectra on the number of laser shots in spectral acquisition, both from an analytical and a biological perspective.

**Fig 4:**
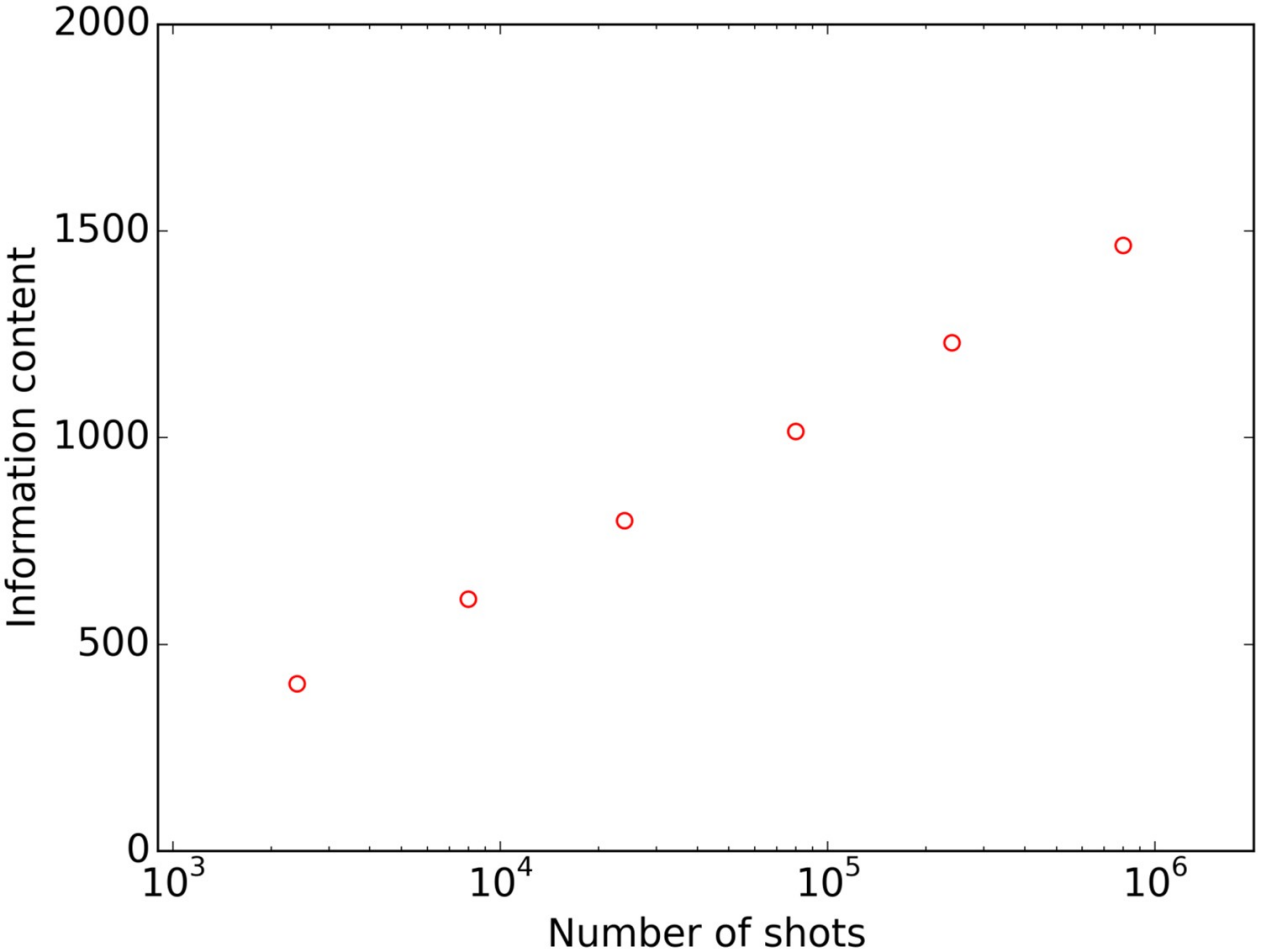
The dependence of the analytical information content of mass spectra on the number of laser shots. Spectra were obtained from the MQS set on the SimulTOF100 instrument, as defined in “Materials and methods”.

The observed reduction of the CVs of features with increasing number of laser shots reflects the decrease of noise-related random errors in the measurements of feature values. As can be seen in Fig 4, this is accompanied by an increase of the information content of spectra. Hence increasing the number of laser shots allows for more reliable differentiation of serum samples.

### Association of peaks with biological processes

The question arises whether the increase in analytical information content with increasing number of laser shots described above leads to an increased ability to detect biologically important phenomena. We address this question in the framework of set enrichment analysis, which estimates the association of individual features (peaks) with a set of biological processes (see “Materials and methods”). We analyzed how this association depends on the number of shots, using spectra up to 400K shots from the BR set. Asking which peaks are associated with a biological process we decide on a p-value cutoff of p<0.01 and set a false discovery rate (FDR) cut-off of 5%. The number of peaks meeting these criteria for different numbers of laser shots and for all investigated biological processes is shown in Table 3. For some processes, e.g. acute inflammatory response, there are many features associated even at low shot numbers (with fluctuations within the FDR), while for other processes, e.g. innate immune response and immune tolerance, the number of associated features increases with shot number indicating that increasing shot number allows for a deeper view of biology in serum profiling.

**Table 3:** Number of peaks associated with the listed biological processes at a p-value cutoff of 0.01 and with a FDR < 5% obtained for the BR set.

Biological Process	8K	24K	80K	240K	400K
Acute inflammatory response	106	105	104	108	109
Acute phase reaction	122	121	119	122	122
Complement activation	58	60	67	70	70
Cytokine production involved in immune response	1	2	3	2	3
Response to hypoxia	0	3	4	7	7
Extracellular matrix organization	0	0	0	0	0
Epithelial- mesenchymal transition	0	0	0	0	0
Angiogenesis	1	2	1	2	2
IFN type 1 signaling/ response	22	26	28	28	33
IFN γ signaling/ response	18	20	27	25	25
Glycolysis	1	1	1	2	2
Behavior	3	6	5	7	8
Cellular component morphogenesis	3	2	3	3	4
Immune tolerance and suppression	22	26	31	28	31
Chronic inflammatory response	0	1	2	1	2
Type 17 immune response	4	4	1	3	2
Innate immune response	2	2	3	6	8
Wound healing	96	102	103	104	100

As this study is devoted to the role of the number of laser shots in MALDI-MS profiling of unfractionated serum, and, in particular, to improvements that can be achieved by increasing the number of shots, we have adopted an approach to analysis of the association of MALDI peaks with biological processes that does not require the assignment of peaks to specific proteins and their fragments. Remarkably, set enrichment analysis approach [75, 87] makes this possible. Data on assignment of some MALDI peaks to specific proteins does exist in the literature [1–3, 33], but most of the peaks that we observe remain unassigned. This is a separate important problem which is outside of the scope of this study, and can be addressed by methods such as tandem mass spectrometry.

## Discussion

We have presented a method for improving the sensitivity of MALDI-TOF mass spectrometry by increasing the signal-to-noise ratio of the measurements leading to an increase in the number of measurable circulating proteins from human serum samples. The same approach can be performed without modification for plasma. We observed that high-frequency noise in the spectra decreased approximately as an inverse square root of the number of shots, all the way up to 10^8^ laser shots. This led to an increase of the observable abundance range to about 4 orders of magnitude (compared to about 2 orders of magnitude for 8000 laser shots) and the number of clearly observable and quantitatively useful peaks in MALDI-TOF mass spectra of unfractionated serum to about 800 peaks.

The extremely high number of laser shots (in the order of millions) presented here is not practical in high throughput operations, and for routine applications one needs to select a number of shots that is practically possible and retains the advantages of using many laser shots. We have decided to use averages generated from 400,000 laser shots for the routine generation of tests to be used in the clinical setting. On the SimulTOF100 mass spectrometer this requires spotting the sample onto eight separate MALDI spots (prepared as described in the section “Materials and methods”) and consumes about 3 μl of serum. Reserving 32 spots for four reference samples, this results in a batch size of 43 samples using a 384 well plate. With current qualified instrument settings it takes about 30 hours to run a batch of 47 samples including the 4 reference samples. For these 400,000 shot spectra the median CV of the CV distributions is 2.31%, the number of observed peaks at a SNR cut-off of 15 is 677 for the reference sample, and the mean number of observed peaks for samples in the machine qualification set at the same SNR cut-off is 646.

In order to achieve the presented decrease in noise, the resulting increase in SNR and in the number of observable peaks, and the corresponding improvement in reproducibility, much care has to be taken, especially with regards to spectral pre-processing. The alignment of the spectra to be averaged is of principal importance because even slight inaccuracies in this part can lead to peak broadening in the averaged spectra, and this loss in resolution limits the number of observable peaks, in addition to the limit imposed by the instrument resolution.

While the results presented here open the theoretical possibility of probing much deeper into the proteome than previously considered possible, they represent an idealized setting. As we randomly sampled raw spectra from acquisitions over many years, batch effects could be ignored. In clinical practice, we do not have a large reservoir of spectra available for individual samples spanning many batches. Instead a sample is prepared and collected within a single batch. To compensate for batch effects, we spot the reference sample at the beginning and at the end of each MALDI plate, and apply additional batch correction processing to map to previously acquired batches serving as baselines for clinical tests. We have established rigorous instrument qualification procedures to minimize batch effects and ensure test reproducibility, based on running a plate containing the MQS set of samples and confirming concordance with the “gold standard” MQS acquisition.

In general, the peaks we observe should be related to proteins of the classical plasma proteome as described in [30], but there could be other proteins visible in certain sample sets that are not usually described in the literature. We could increase the mass range of our measurements beyond the 3-30kDa range to further extend the number of detected proteins with this method. However, in the high mass range the resolution of the MALDI TOF instruments we used in this study becomes very poor. In the m/z range 30–70 kDa we have observed only a handful of very broad peaks. Thus we decided to limit the m/z range in this study to 30 kDa. In the low mass region highly variable metabolomic decay products may confound our ability to reliably detect peptides.

## Conclusions

The results demonstrate that increasing the number of laser shots increases the number of measurable peaks in human serum samples without requiring fractionation steps. This holds true over a large dynamic range and appears to be limited by instrument resolution rather than sensitivity.

The approach requires only very small amounts of serum or plasma, less than 5 μl, which preserves clinical sample pools from retrospective studies. The reproducibility of the method compares well with multiplexed techniques, which typically show CVs between > 4% and < 15% [4,6,8], with exception of the aptamer-based SOMAscan assay, which shows median CV about 3–4% [13]. The method does not require multiple dilution steps, which are often needed when the population variation of the abundance of the chosen proteins is large [4–13]. In addition, this method allows to separately measure different splice isoforms or post translationally modified proteins that may have different biological functions [14–20].

Increasing the number of laser shots leads to an increase of information content, both from an analytical and biological perspective. Assuming that subtle questions related to drug efficacy and toxicity, especially in oncology in the era of immunotherapy and early detection, require the detection and measurement of complicated regulatory processes, it is possible that the presented approach can lead to more reliable test discoveries, especially in the context of multivariate tests using modern machine learning methods.

This approach has been successfully applied to multiple test development efforts related to the development of prognostic and predictive tests in the area of oncology. Of particular relevance are the validated results obtained for a pre-treatment test identifying patients with metastatic cancer who are resistant to checkpoint inhibition [90–94]. Immune oncology should be a fertile ground for multivariate methods investigating the circulating proteome, given the interplay between tumor biology and the host immune system.

In summary, we have presented a method that significantly increases the useful information that can be mined from mass spectrometry-based profiling of serum samples. The method extends the observable dynamic range in a single workflow on MALDI-TOF platforms and could lead to the development of many more clinically useful and validated tests.

## Supporting information

S1 Appendix Supplementary materials. Sample Preparation and Spectral Acquisition for Bruker Ultraflextreme and for SimulTOF100. The list of features used for the reproducibility analysis and the concordance analysis of the machine qualification set. The list of biological processes used in the set enrichment analysis.
(PDF)

S1 Fig Effect of the peak width on the number of detected peaks. A) Average spectrum acquired on SimulTOF100, 100 million laser shots. 62 peaks are detected in the m/z range shown. B) The same spectrum with resolution artificially reduced by a factor of 2 by applying a moving average filter. The number of detected peaks is decreased to 32. The signal/noise ratio threshold for peak detection (SNR = 10) is the same in both A and B.
(TIF)

S2 Fig Average spectrum used to compute the peak density depicted in Fig 2C. The 100 million shot spectrum is plotted as a function of m/z. Detected peaks are marked by vertical lines.
(DOCX)

S1 File Spectrum file: Average spectrum used to compute the peak density depicted in Fig 2C. The 100 million shot spectrum in a 2-column (m/z, intensity) text format.
(ZIP)

## References

[1] Karpova MA, Moshkovskii SA, Toropygin IY, Archakov AI. Cancer-specific MALDI-TOF profiles of blood serum and plasma: biological meaning and perspectives. J Proteomics 2010; 73(3): 537–551. 10.1016/j.jprot.2009.09.011 19782778

[2] Tiss A, Smith C, Menon U, Jacobs I, Timms JF, Cramer R, A well-characterised peak identification list of MALDI MS profile peaks for human blood serum. Proteomics 2010; 10(18):3388–3392. 10.1002/pmic.201000100 20707003

[3] Pietrowska M, Widłak P. MALDI-MS-Based Profiling of Serum Proteome: Detection of Changes Related to Progression of Cancer and Response to Anticancer Treatment. Int J Proteomics 2012; 2012: 926427 10.1155/2012/926427 22900176 PMC3413974

[4] duPont NC, Wang K, Wadhwa PD, Culhane JF, Nelson EL. Validation and comparison of luminex multiplex cytokine analysis kits with ELISA: Determinations of a panel of nine cytokines in clinical sample culture supernatants. J Reprod Immunol. 2005; 66(2): 175–191. 10.1016/j.jri.2005.03.005 16029895 PMC5738327

[5] Elshal MF, McCoy JP. Multiplex bead array assays: performance evaluation and comparison of sensitivity to ELISA. Methods 2006; 38(4): 317–323. 10.1016/j.ymeth.2005.11.010 16481199 PMC1534009

[6] Dossus L, Becker S, Achaintre D, Kaaks R, Rinaldi S. Validity of multiplex-based assays for cytokine measurements in serum and plasma from “non-diseased” subjects: Comparison with ELISA. J Immunol Methods 2009; 350(1–2): 125–132. 10.1016/j.jim.2009.09.001 19748508

[7] Perkel JM. Multiplexed Protein Assays. 28 March 2011 [cited 20 March 2018] https://www.biocompare.com/Editorial-Articles/41806-Multiplexed-Protein-Assays/

[8] Tighe PJ, Ryder RR, Todd I, Fairclough LC. ELISA in the multiplex era: Potentials and pitfalls. Proteomics Clin. Appl. 2015; 9(3–4):406–422. 10.1002/prca.201400130 25644123 PMC6680274

[9] Ellington AD, Szostak JW. In vitro selection of RNA molecules that bind specific ligands. Nature 1990; 346(6287): 818–822. 10.1038/346818a0 1697402

[10] Tuerk C, Gold L. Systematic evolution of ligands by exponential enrichment: RNA ligands to bacteriophage T4 DNA polymerase. Science 1990; 249(4968): 505–510. 10.1126/science.2200121 2200121

[11] Gold L, Janjic N, Jarvis T, Schneider D, Walker JJ, Wilcox SK, Zichi D. Aptamers and the RNA world, past and present. Cold Spring Harb Perspect Biol. 2012; 4(3): a003582 10.1101/cshperspect.a003582 21441582 PMC3282410

[12] Gold L, Ayers D, Bertino J, Bock C, Bock A, Brody EN, et al Aptamer-Based Multiplexed Proteomic Technology for Biomarker Discovery. PLoS One 2010; 5(12): e15004 10.1371/journal.pone.0015004 21165148 PMC3000457

[13] Candia J, Cheung F, Kotliarov Y, Fantoni G, Sellers B, Griesman T, et al Assessment of Variability in the SOMAscan Assay. Sci Rep. 2017; 7(1): 14248 10.1038/s41598-017-14755-5 29079756 PMC5660188

[14] Nedelkov D, Kiernan UA, Niederkofler EE, Tubbs KA, Nelson RW. Investigating diversity in human plasma proteins. PNAS 2005; 102(31): 10852–10857. 10.1073/pnas.0500426102 16043703 PMC1180507

[15] Trenchevska O, Nelson RW, Nedelkov D. Mass Spectrometric Immunoassays in Characterization of Clinically Significant Proteoforms. Proteomes 2016; 4(1): 13 10.3390/proteomes4010013 28248223 PMC5217360

[16] Trenchevska O, Nelson RW, Nedelkov D. Mass spectrometric immunoassays for discovery, screening and quantification of clinically relevant proteoforms. Bioanalysis 2016; 8(15) 10.4155/bio-2016-0060 27396364 PMC4964736

[17] Nedelkov D. Human proteoforms as new targets for clinical mass spectrometry protein tests. Expert Review of Proteomics, 2017; 14(8): 691–699. 10.1080/14789450.2017.1362337 28756725

[18] Wu DC, Wang KY, Wang SSW, Huang CM, Lee YW, Chen MI, et al Exploring the expression bar code of SAA variants for gastric cancer detection. Proteomics 2017; 17(11): 1600356 10.1002/pmic.201600356 28493537

[19] Kiernan UA, Tubbs KA, Nedelkov D, Niederkofler EE, Nelson RW. Detection of novel truncated forms of human serum amyloid A protein in human plasma. FEBS Letters 2003; 537(1–3): 166–170. 10.1016/s0014-5793(03)00097-8 12606051

[20] Yassine HN, Trenchevska O, He H, Borges CR, Nedelkov D, Mack W, et al Serum Amyloid A Truncations in Type 2 Diabetes Mellitus. PLoS ONE 2015; 10(1): e0115320 10.1371/journal.pone.0115320 25607823 PMC4301920

[21] Dakna M, Harris K, Kalousis A, Carpentier S, Kolch W, Schanstra JP, et al Addressing the challenge of defining valid proteomic biomarkers and classifiers. BMC Bioinformatics 2010; 11: 594 10.1186/1471-2105-11-594 21208396 PMC3017845

[22] Kolch W, Neususs C, Pelzing M, Mischak H. Capillary electrophoresis-mass spectrometry as a powerful tool in clinical diagnosis and biomarker discovery. Mass Spectrom Rev 2005; 24(6): 959–977. 10.1002/mas.20051 15747373

[23] Listgarten J, Emili A. Statistical and computational methods for comparative proteomic profiling using liquid chromatography-tandem mass spectrometry. Mol Cell Proteomics. 2005; 4(4): 419–34. 10.1074/mcp.R500005-MCP200 15741312

[24] Swan AL, Mobasheri A, Allaway D, Liddell S, Bacardit J. Application of machine learning to proteomics data: classification and biomarker identification in postgenomics biology. OMICS. 2013; 17(12): 595–610. 10.1089/omi.2013.0017 24116388 PMC3837439

[25] Robotti E, Manfredi M, Marengo E. Biomarkers Discovery through Multivariate Statistical Methods: A Review of Recently Developed Methods and Applications in Proteomics. J Proteomics Bioinform 2014; S3: 003 10.4172/jpb.S3-003

[26] Fan Z, Kong F, Zhou Y, Chen Y, Dai Y. Intelligence Algorithms for Protein Classification by Mass Spectrometry. Biomed Res Int. 2018; 2018: 2862458 10.1155/2018/2862458 30534555 PMC6252195

[27] Grapov D, Fahrmann J, Wanichthanarak K, Khoomrung S. Rise of Deep Learning for Genomic, Proteomic, and Metabolomic Data Integration in Precision Medicine. OMICS. 2018; 22(10): 630–636. 10.1089/omi.2018.0097 30124358 PMC6207407

[28] Roder J, Oliveira C, Net L, Tsypin M, Linstid B, Roder H. A dropout-regularized classifier development approach optimized for precision medicine test discovery from omics data. BMC Bioinformatics. 2019; 20(1): 325 10.1186/s12859-019-2922-2 31196002 PMC6567499

[29] Roder H, Oliveira C, Net L, Linstid B, Tsypin M, Roder J. Robust identification of molecular phenotypes using semi-supervised learning. BMC Bioinformatics. 2019; 20(1): 273 10.1186/s12859-019-2885-3 31138112 PMC6540576

[30] Anderson NL, Anderson NG. The human plasma proteome: History, character, and diagnostic prospects. Mol. Cell. Proteomics 2002; 1(11): 845–867. 10.1074/mcp.r200007-mcp200 12488461

[31] Service RF. Proteomics ponders prime time. Science 2008; 321: 1758–1761. 10.1126/science.321.5897.1758 18818332

[32] Schwenk JM, Omenn GS, Sun Z, Campbell DS, Baker MS, Overall CM, et al The Human Plasma Proteome Draft of 2017: Building on the Human Plasma PeptideAtlas from Mass Spectrometry and Complementary Assays. J Proteome Res 2017; 16(12): 4299–4310. 10.1021/acs.jproteome.7b00467 28938075 PMC5864247

[33] Hortin GL. The MALDI-TOF mass spectrometric view of the plasma proteome and peptidome. Clin Chem. 2006; 52(7): 1223–37. 10.1373/clinchem.2006.069252 16644871

[34] Greco V, Piras C, Pieroni L, Ronci M, Putignani L, Roncada P, et al Applications of MALDI-TOF mass spectrometry in clinical proteomics. Expert Rev Proteomics. 2018; 15(8): 683–696. 10.1080/14789450.2018.1505510 30058389

[35] Krutchinsky AN, Chait BT. On the nature of the chemical noise in MALDI mass spectra. J Am Soc Mass Spectrom. 2002; 13(2): 129–34. 10.1016/s1044-0305(01)00336-1 11838016

[36] Knochenmuss R, Karbach V, Wiesli U, Breuker K, Zenobi R. The Matrix Suppression Effect in Matrix Assisted Laser Desorption/Ionization: Application to Negative Ions and Further Characteristics. Rapid Commun. Mass Spectrom. 1998; 12(9): 529–534.

[37] Burkitt WI, Giannakopulos AE, Sideridou F, Bashir S, Derrick PJ. Discrimination effects in MALDI-MS of mixtures of peptides—Analysis of the Proteome. Aust J Chem 2003; 56 (5): 369–377

[38] Luxembourg SL, McDonnell LA, Duursma MC, Guo X, Heeren RMA. Effect of Local Matrix Crystal Variations in Matrix-Assisted Ionization Techniques for Mass Spectrometry. Anal. Chem. 2003; 75(10): 2333–2341. 10.1021/ac026434p 12918974

[39] Jones EA, Lockyer NP, Kordys J, Vickerman JC. Suppression and Enhancement of Secondary Ion Formation Due to the Chemical Environment in Static-Secondary Ion Mass Spectrometry. J. Am. Soc. Mass Spectrom. 2007; 18(8): 1559–1567. 10.1016/j.jasms.2007.05.014 17604641

[40] Aresta A, Calvano CD, Palmisano F, Zambonin CG, Monaco A, Tommasi S, et al Impact of sample preparation in peptide/protein profiling in human serum by MALDI-TOF mass spectrometry. J Pharm Biomed Anal. 2008; 46(1): 157–164. 10.1016/j.jpba.2007.10.015 18035512

[41] Weidmann S, Mikutis G, Barylyuk K, Zenobi R. Mass discrimination in high-mass MALDI-MS. J Am Soc Mass Spectrom. 2013; 24(9): 1396–404. 10.1007/s13361-013-0686-x 23836380

[42] Albrethsen J. Reproducibility in protein profiling by MALDI-TOF mass spectrometry. Clin Chem. 2007; 53(5): 852–8. 10.1373/clinchem.2006.082644 17395711

[43] Rose K, Bougueleret L, Baussant T, Bohm G, Botti P, Colinge J, et al Industrial scale proteomics: from liters of plasma to chemically synthesized proteins. Proteomics 2004; 4(7): 2125–2150. 10.1002/pmic.200300718 15221774

[44] Metz TO, Jacobs JM, Gritsenko MA, Fontès G, Qian WJ, Camp DG, et al Advances and challenges in liquid chromatography-mass spectrometry-based proteomics profiling for clinical applications. Mol Cell Proteomics 2006; 5(10): 1727–1744. 10.1074/mcp.M600162-MCP200 16887931 PMC1781927

[45] Dayon L, Kussmann M. Proteomics of human plasma: A critical comparison of analytical workflows in terms of effort, throughput and outcome. EuPA Open Proteomics 2013; 1: 8–16. 10.1016/j.euprot.2013.08.001

[46] Li XJ, Lee LW, Hayward C, Brusniak MY, Fong PY, McLean M, et al An integrated quantification method to increase the precision, robustness, and resolution of protein measurement in human plasma samples. Clin Proteomics. 2015; 12(1): 3 10.1186/1559-0275-12-3 25838814 PMC4363461

[47] Cominetti O, Núñez Galindo A, Corthésy J, Oller Moreno S, Irincheeva I, Valsesia A, et al Proteomic Biomarker Discovery in 1000 Human Plasma Samples with Mass Spectrometry. J Proteome Res. 2016 2 5;15(2):389–99. 10.1021/acs.jproteome.5b00901 26620284

[48] Keshishian H, Burgess MW, Specht H, Wallace L, Clauser KR, Gillette MA, et al Quantitative, multiplexed workflow for deep analysis of human blood plasma and biomarker discovery by mass spectrometry. Nat Protoc. 2017; 12(8): 1683–1701. 10.1038/nprot.2017.054 28749931 PMC6057147

[49] Dayon L, Núñez Galindo A, Cominetti O, Corthésy J, Kussmann M. A Highly Automated Shotgun Proteomic Workflow: Clinical Scale and Robustness for Biomarker Discovery in Blood. Methods Mol Biol. 2017; 1619: 433–449. 10.1007/978-1-4939-7057-5_30 28674902

[50] Bhosale SD, Moulder R, Kouvonen P, Lahesmaa R, Goodlett DR Mass Spectrometry-Based Serum Proteomics for Biomarker Discovery and Validation. Methods Mol Biol. 2017; 1619: 451–466. 10.1007/978-1-4939-7057-5_31 28674903

[51] Bruderer R, Muntel J, Müller S, Bernhardt OM, Gandhi T, Cominetti O, et al Analysis of 1508 Plasma Samples by Capillary-Flow Data-Independent Acquisition Profiles Proteomics of Weight Loss and Maintenance. Mol Cell Proteomics. 2019; 18(6): 1242–1254. 10.1074/mcp.RA118.001288 30948622 PMC6553938

[52] Pernemalm M, Sandberg A, Zhu Y, Boekel J, Tamburro D, Schwenk JM, et al In-depth human plasma proteome analysis captures tissue proteins and transfer of protein variants across the placenta. Elife. 2019; 8: e41608 10.7554/eLife.41608 30958262 PMC6519984

[53] Anderson L, Hunter CL. Quantitative mass spectrometric multiple reaction monitoring assays for major plasma proteins. Mol Cell Proteomics 2006; 5(4): 573–588. 10.1074/mcp.M500331-MCP200 16332733

[54] Addona TA, Abbatiello SE, Schilling B, Skates SJ, Mani DR, Bunk DM, et al Multi-site assessment of the precision and reproducibility of multiple reaction monitoring-based measurements of proteins in plasma. Nat Biotechnol 2009; 27(7): 633–641. 10.1038/nbt.1546 19561596 PMC2855883

[55] Chambers AG, Percy AJ, Yang J, Borchers CH. Multiple Reaction Monitoring Enables Precise Quantification of 97 Proteins in Dried Blood Spots. Mol Cell Proteomics 2015; 14(11): 3094–3104. 10.1074/mcp.O115.049957 26342038 PMC4638049

[56] Ozcan S, Cooper JD, Lago SG, Kenny D, Rustogi N, Stocki P, Bahn S. Towards reproducible MRM based biomarker discovery using dried blood spots. Sci Rep 2017; 7: 45178 10.1038/srep45178 28345601 PMC5366927

[57] Lehmann S, Picas A, Tiers L, Vialaret J, Hirtz C. Clinical perspectives of dried blood spot protein quantification using mass spectrometry methods. Crit Rev Clin Lab Sci. 2017; 54(3): 173–184. 10.1080/10408363.2017.1297358 28393579

[58] Li H, Han J, Pan J, Liu T, Parker CE, Borchers CH. Current trends in quantitative proteomics—an update. J Mass Spectrom 2017; 52(5): 319–341. 10.1002/jms.3932 28418607

[59] Kearney P, Hunsucker SW, Li XJ, Porter A, Springmeyer S, Mazzone P. An integrated risk predictor for pulmonary nodules. PLoS One 2017; 12(5): e0177635 10.1371/journal.pone.0177635 28545097 PMC5435179

[60] Silvestri GA, Tanner NT, Kearney P, Vachani A, Massion PP, Porter A, et al Assessment of Plasma Proteomics Biomarker’s Ability to Distinguish Benign From Malignant Lung Nodules: Results of the PANOPTIC (Pulmonary Nodule Plasma Proteomic Classifier) Trial. Chest 2018; 154(3): 491–500. 10.1016/j.chest.2018.02.012 29496499 PMC6689113

[61] Römpp A, Dekker L, Taban I, Jenster G, Boogerd W, Bonfrer H, et al Identification of leptomeningeal metastasis-related proteins in cerebrospinal fluid of patients with breast cancer by a combination of MALDI-TOF, MALDI-FTICR and nanoLC-FTICR MS. Proteomics 2007; 7(3): 474–81. 10.1002/pmic.200600719 17274072

[62] Stoop MP, Dekker LJ, Titulaer MK, Lamers RJ, Burgers PC, Sillevis Smitt PA, et al Quantitative matrix-assisted laser desorption ionization-fourier transform ion cyclotron resonance (MALDI-FT-ICR) peptide profiling and identification of multiple-sclerosis-related proteins. J Proteome Res. 2009; 8(3): 1404–14. 10.1021/pr8010155 19159215

[63] Nicolardi S, Palmblad M, Hensbergen PJ, Tollenaar RA, Deelder AM, van der Burgt YE. Precision profiling and identification of human serum peptides using Fourier transform ion cyclotron resonance mass spectrometry. Rapid Commun Mass Spectrom. 2011; 25(23): 3457–63. 10.1002/rcm.5246 22095492

[64] Nicolardi S, van der Burgt YE, Wuhrer M, Deelder AM. Mapping O-glycosylation of apolipoprotein C-III in MALDI-FT-ICR protein profiles. Proteomics. 2013; 13(6): 992–1001. 10.1002/pmic.201200293 23335445

[65] Nicolardi S. Development of ultrahigh resolution FTICR mass spectrometry methods for clinical proteomics. Doctoral Dissertation, Leiden University. 2014. ISBN: 978-94-6182-435-6. http://hdl.handle.net/1887/25784

[66] Nicolardi S, Bogdanov B, Deelder AM, Palmblad M, van der Burgt YE. Developments in FTICR-MS and Its Potential for Body Fluid Signatures. Int J Mol Sci. 2015; 16(11): 27133–44. 10.3390/ijms161126012 26580595 PMC4661870

[67] Yergey AL, Coorssen JR, Backlund PS Jr, Blank PS, Humphrey GA, Zimmerberg J, et al De novo sequencing of peptides using MALDI/TOF-TOF. J Am Soc Mass Spectrom 2002; 13(7): 784–791. 10.1016/S1044-0305(02)00393-8 12148803

[68] Vestal ML, Campbell JM. Tandem time-of-flight mass spectrometry. Methods Enzymol. 2005; 402: 79–108. 10.1016/S0076-6879(05)02003-3 16401507

[69] Vestal ML. Modern MALDI time-of-flight mass spectrometry. J Mass Spectrom. 2009; 44(3): 303–17. 10.1002/jms.1537 19142962

[70] Vestal ML. The future of biological mass spectrometry. J Am Soc Mass Spectrom 2011; 22(6): 953–9. 10.1007/s13361-011-0108-x 21953036

[71] Standing KG, Vestal ML. Time-of-flight mass spectrometry (TOFMS): From niche to mainstream. International Journal of Mass Spectrometry 2015; 377: 295–308. 10.1016/j.ijms.2014.09.002

[72] Mitchell M, Mali S, King CC, Bark SJ. Enhancing MALDI Time-Of-Flight Mass Spectrometer Performance through Spectrum Averaging. PLoS ONE 2015; 10(3): e0120932 10.1371/journal.pone.0120932 25798583 PMC4370844

[73] Jansen BC, Bondt A, Reiding KR, Lonardi E, De Jong CJ, Falck D, et al Pregnancy-associated serum N-glycome changes studied by high-throughput MALDI-TOF-MS. Sci Rep. 2016; 6: 23296 10.1038/srep23296 27075729 PMC4831011

[74] Reiding KR, Blank D, Kuijper DM, Deelder AM, Wuhrer M. High-throughput profiling of protein N-glycosylation by MALDI-TOF-MS employing linkage-specific sialic acid esterification. Anal Chem. 2014; 86(12): 5784–93. 10.1021/ac500335t 24831253

[75] Subramanian A, Tamayo P, Mootha VK, Mukherjee S, Ebert BL, Gillette MA, et al Gene set enrichment analysis: a knowledge-based approach for interpreting genome-wide expression profiles. Proc Natl Acad Sci USA. 2005; 102(43): 15545–50. 10.1073/pnas.0506580102 16199517 PMC1239896

[76] Andrew AM. Another efficient algorithm for convex hulls in two dimensions. Information Processing Letters 1979; 9(5):216–219. 10.1016/0020-0190(79)90072-3

[77] Algorithm Implementation/Geometry/Convex hull/Monotone chain. [cited 28 August 2017]. https://en.wikibooks.org/wiki/Algorithm_Implementation/Geometry/Convex_hull/Monotone_chain

[78] Gibb S, Strimmer K. Mass Spectrometry Analysis Using MALDIquant In: Datta S, Mertens BJA, editors. Statistical Analysis of Proteomics, Metabolomics, and Lipidomics Data Using Mass Spectrometry. Frontiers in Probability and the Statistical Sciences, Springer International Publishing Switzerland 2017, pp.101–124. 10.1007/978-3-319-45809-0_6

[79] Savitzky A, Golay MJE. Smoothing and Differentiation of Data by Simplified Least Squares Procedures. Anal Chem. 1964; 36(8): 1627–1639. 10.1021/ac60214a047

[80] Inverse Erf. [cited 01 October 2019]. http://mathworld.wolfram.com/InverseErf.html

[81] Gene Ontology Consortium: http://www.geneontology.org/

[82] Ashburner M, Ball CA, Blake JA, Botstein D, Butler H, Cherry JM, et al Gene ontology: tool for the unification of biology. The Gene Ontology Consortium. Nat Genet. 2000; 25(1): 25–29. 10.1038/75556 10802651 PMC3037419

[83] Carbon S, Ireland A, Mungall CJ, Shu S, Marshall B, Lewis S, et al AmiGO: online access to ontology and annotation data. Bioinformatics. 2009; 25(2): 288–289. 10.1093/bioinformatics/btn615 19033274 PMC2639003

[84] http://amigo.geneontology.org/amigo.

[85] https://www.ebi.ac.uk/QuickGO/.

[86] Guide to GO evidence codes. http://geneontology.org/docs/guide-go-evidence-codes/

[87] Grigorieva J, Asmellash S, Oliveira C, Roder H, Net L, Roder J. Application of protein set enrichment analysis to correlation of protein functional sets with mass spectral features and multivariate proteomic tests. Clinical Mass Spectrometry 2019; (Forthcoming) 10.1016/j.clinms.2019.09.001

[88] Roder J, Linstid B, Oliveira C. Improving the power of gene set enrichment analyses. BMC Bioinformatics. 2019; 20(1): 257 10.1186/s12859-019-2850-1 31101008 PMC6525372

[89] Benjamini Y, Hochberg Y. Controlling the False Discovery Rate: A Practical and Powerful Approach to Multiple Testing. Journal of the Royal Statistical Society. Series B (Methodological) 1995; 57(1): 289–300.

[90] Weber JS, Sznol M, Sullivan RJ, Blackmon S, Boland G, Kluger HM, et al A Serum Protein Signature Associated with Outcome after Anti-PD-1 Therapy in Metastatic Melanoma. Cancer Immunol Res. 2018; 6(1): 79–86. 10.1158/2326-6066.CIR-17-0412 29208646

[91] Smit EF, Aerts JG, Muller M, Niemeijer AN, Roder H, Oliveira C, et al Prediction of primary resistance to anti-PD1 therapy (APD1) in second-line NSCLC. In: 43rd ESMO Congress (ESMO 2018) 19–23 October 2018, Munich, Germany. Annals of Oncology 2018; 29(suppl_8): mdy269.068.

[92] Aerts J, Smit E, Muller M, Niemeijer A, Oliveira C, Roder H, et al Detection of Primary Immunotherapy Resistance to PD-1 Checkpoint Inhibitors (PD1CI) in 2nd Line NSCLC. In: IASLC 19th World Conference on Lung Cancer, 23–26 September 2018, Toronto, Canada. J Thorac Oncol. 2018; 13(10): S424.

[93] Kowanetz M, Leng N, Roder J, Oliveira C, Asmellash S, Meyer K, et al Evaluation of Immune–related Markers in circulating proteome and their association with atezolizumab efficacy in patients with 2L+ NSCLC. In: 33rd Annual Meeting of the Society for Immunotherapy of Cancer (SITC 2018), 8–11 November 2018, Washington DC, USA. J Immunother Cancer. 2018; 6(Suppl 1): 114 10.1186/s40425-018-0422-y 30400835 PMC6220480

[94] Ascierto PA, Capone M, Grimaldi AM, Mallardo D, Simeone E, Madonna G, et al Proteomic test for anti-PD-1 checkpoint blockade treatment of metastatic melanoma with and without BRAF mutations. J Immunother Cancer. 2019; 7(1): 91 10.1186/s40425-019-0569-1 30925943 PMC6440152

