# Supplementary material for: Extending the information content of the MALDI analysis of biological fluids via multi-million shot analysis
Source: PLoS One. 2019 Dec 9;14(12):e0226012. doi: 10.1371/journal.pone.0226012 (PMC6901224; doi:10.1371/journal.pone.0226012)
Supplement: S1 Appendix — Sample Preparation and Spectral Acquisition for Bruker Ultraflextreme and for SimulTOF100. The list of features used for the reproducibility analysis and the concordance analysis of the machine qualification set. The list of biological processes used in the set enrichment analysis. (PDF) [file pone.0226012.s001.pdf]

Supplementary Materials

Sample Preparation and Spectral Acquisition

Bruker Ultraflextreme

Serum samples were thawed, diluted 1:10 in water and mixed with an equal volume of freshly prepared matrix solution (sinapinic acid in acetonitrile/water 50:50 v/v containing 0.1% trifluoroacetic acid). The serum:matrix mix was directly spotted onto a polished stainless steel MALDI target plate (Bruker Daltonics, Bremen, Germany). The target plates were then placed in the mass spectrometer for spectral acquisition.

The Ultraflextreme used is equipped with a Smartbeam 2 laser, delayed-extraction electronics, and a LeCroy LSA2000 digitizer controlled by Bruker's Flexcontrol™ 3.4 software. The automated acquisition method in Flexcontrol was modified to acquire and save multiple sub-spectra (800 shot spectra) from each MALDI spot. Spectra in the mass-to-charge ratio (m/z) range of 3,000 to 30,000 Daltons were acquired in linear positive ion mode with delayed extraction. The laser repetition rate was set at 2000Hz and the laser power adjusted to just above threshold for ion production. The instrument was externally calibrated with a calibration mix (Protein Calibration Standard I) from Bruker.

Each preparation of serum plus matrix was spotted onto 8-10 MALDI spots and 800 shot spectra were collected from 200-250 locations or rasters per spot. Each 800 shot spectrum generated from one of the individual rasters was saved as a separate spectrum file. The spectral files were converted to ASCII for further processing.

SimulTOF100

On the SimulTOF100 a different sample preparation method was used in order to more closely mimic the workflow used in clinical experiments. Serum samples were thawed and 3 µl aliquots of each sample spotted onto serum cards. The cards were allowed to dry for 1 hour at ambient temperature after which the whole serum spot was punched out with a 6mm skin biopsy punch. Each punch was placed in a centrifugal filter with 0.45 µm nylon membrane (VWR). One hundred microliters of HPLC grade water (JT Baker) was added to the centrifugal filter containing the punch. The punches were vortexed gently for 10 minutes then spun down at 14,000 rcf for 2 minutes. The flow-through was removed and transferred back on to the punch for a second round of extraction. For the second round of extraction, the punches were vortexed gently for 3 minutes then spun down at 14,000 rcf for 2 minutes. Twenty microliters of the filtrate from each sample was then transferred to a 0.5 ml eppendorf tube for MALDI analysis.

All subsequent sample preparation steps were carried out in a custom designed humidity and temperature control chamber (Coy Lab Products, Grass Lake, MI, USA). The temperature was set to 30 °C and the relative humidity to 10%.

An equal volume of freshly prepared matrix (25 mg of sinapinic acid per 1 ml of 50% acetonitrile: 50% water plus 0.1% TFA) was added to each 20µl serum extract and the mix vortexed for 30 sec. The first three aliquots (3 x 2µl) of sample:matrix mix were discarded. Eight aliquots of 2µl sample:matrix mix were then spotted onto a stainless steel MALDI target plate (SimulTOF Systems). The MALDI target was allowed to dry in the chamber before placement in the MALDI mass spectrometer.

MALDI spectra were obtained using a SimulTOF100 MALDI-TOF mass spectrometer (SimulTOF Systems, Marlborough, MA, USA). The instrument was set to operate in positive ion mode, with ions generated using a 349 nm, diode-pumped, frequency-tripled Nd:YLF laser operated at a laser repetition rate of 0.5 kHz.

External calibration was performed using a mixture of standard proteins (Bruker Daltonics, Germany) consisting of insulin (*m/z* =5734.51 Da), ubiquitin (*m/z* = 8565.76 Da), cytochrome C (*m/z* = 12360.97 Da), and myoglobin (*m/z* = 16952.30 Da) or serum peaks present in the reference sample (mz = 3320, 4158.73, 6636.79 , 9429.30, 13890.44, 15877.58, 28093.95 Da).

Spectra from each MALDI spot were collected as 800 shot spectra over a mass range of 3 to 75 kDa (only the range from 3 to 30 kDa was used in this analysis) and were 'hardware averaged' as the laser fires continuously across the spot while the stage is moving at a speed of 0.25 mm/sec. A minimum intensity threshold of 0.01 V was used to discard any 'flat line' spectra. All 800 shot spectra with intensity above this threshold were acquired without any further processing.

Features used for the reproducibility analysis of the concordance analysis of the machine qualification set

Left M/Z	Right M/Z
3071.22	3099.16
3099.64	3122.77
3124.22	3164.21
3192.34	3229.04
3231.00	3256.07
3296.71	3348.26
3348.74	3378.62
3379.10	3402.22
3402.71	3433.54
3434.51	3453.30
3454.26	3471.61
3486.54	3521.23
3530.87	3575.20
3583.14	3603.05
3671.81	3693.62
3694.82	3713.28
3713.38	3733.58
3759.25	3786.23
3786.27	3804.76
3805.44	3830.83

3832.00	3851.28
3877.78	3898.49
3899.28	3915.58
3915.70	3939.80
3940.41	3964.50
3995.34	4023.77
4024.14	4038.87
4039.25	4063.54
4080.14	4109.53
4112.17	4126.57
4127.25	4139.52
4165.92	4175.73
4198.95	4222.68
4252.63	4272.38
4272.87	4302.74
4322.97	4350.92
4351.40	4368.27
4369.23	4390.91
4392.36	4425.60
4426.15	4440.90
4445.65	4479.39
4493.06	4519.07
4539.60	4553.09
4553.30	4577.84
4578.09	4602.88
4610.62	4633.26
4633.75	4651.09
4668.35	4680.16
4680.32	4692.43
4694.45	4733.92
4745.60	4764.92
4767.31	4779.75
4780.02	4798.84
4801.90	4834.18
4836.11	4877.54
4878.03	4905.97
4927.53	4943.81
4948.65	4969.62
4970.09	4985.31
4986.71	5011.42
5012.50	5031.62
5033.64	5048.71
5049.07	5085.21

5087.13	5121.83
5121.89	5133.85
5134.35	5161.82
5164.71	5189.28
5211.23	5236.09
5263.48	5312.62
5330.75	5350.64
5351.59	5373.12
5395.67	5418.40
5419.38	5437.02
5442.72	5456.36
5503.42	5535.22
5537.82	5560.82
5561.13	5579.78
5666.47	5683.10
5684.19	5699.72
5699.88	5711.22
5714.46	5726.02
5726.03	5742.81
5767.79	5786.53
5803.89	5816.27
5816.42	5829.11
5832.02	5848.89
5856.95	5879.55
5879.59	5897.90
5898.07	5921.47
5923.79	5943.21
5943.39	5962.13
5973.59	5996.91
5998.01	6019.14
6019.87	6034.75
6035.30	6050.36
6067.08	6095.18
6095.91	6120.52
6186.65	6202.25
6202.49	6217.23
6217.50	6237.52
6275.16	6293.14
6293.98	6312.97
6321.46	6343.87
6346.72	6366.05
6378.77	6407.42
6407.61	6468.77

6470.60	6504.03
6521.11	6548.85
6575.85	6602.67
6603.58	6702.22
6715.26	6745.93
6798.46	6819.77
6825.83	6849.52
6849.89	6868.99
6869.17	6911.42
6911.60	6930.34
6931.26	6963.03
6963.58	6978.64
6979.01	7011.52
7012.07	7030.07
7030.26	7039.99
7040.36	7066.62
7066.99	7084.26
7118.24	7169.66
7178.66	7199.97
7231.75	7254.15
7254.70	7283.36
7283.91	7309.99
7310.54	7372.07
7375.19	7404.95
7405.50	7431.76
7433.23	7460.97
7461.89	7481.36
7524.34	7550.05
7603.32	7631.42
7639.92	7662.13
7704.86	7721.84
7729.99	7742.42
7742.83	7759.00
7760.24	7775.30
7776.52	7801.31
7811.17	7843.39
7871.88	7890.53
7905.09	7923.09
7984.80	8005.01
8006.66	8030.72
8131.01	8175.09
8192.54	8238.82
8239.93	8269.86

8307.15	8352.70
8353.43	8375.48
8378.60	8403.95
8404.50	8423.41
8423.60	8442.33
8457.39	8471.97
8472.10	8486.16
8495.33	8520.09
8520.60	8543.32
8555.29	8574.94
8575.31	8608.74
8650.07	8673.76
8719.85	8729.14
8757.15	8785.43
8800.49	8853.03
8860.56	8882.96
8883.70	8905.92
8906.29	8957.90
8966.17	8987.29
8988.02	9009.33
9009.88	9030.64
9031.74	9051.58
9056.50	9068.30
9068.96	9088.18
9089.96	9106.31
9113.47	9171.51
9196.31	9219.45
9233.96	9254.72
9254.90	9270.88
9271.06	9291.45
9308.35	9331.31
9345.77	9386.64
9387.10	9410.29
9411.21	9496.84
9554.47	9592.35
9613.48	9639.65
9640.11	9669.27
9688.55	9758.58
9779.01	9802.89
9843.02	9889.11
9903.45	9965.21
10067.77	10087.97
10088.18	10111.66

10126.38	10151.18
10151.41	10172.07
10172.76	10196.87
10200.08	10224.19
10224.65	10247.38
10249.52	10274.94
10276.56	10294.77
10295.62	10315.75
10334.89	10359.76
10435.64	10465.26
10466.56	10487.33
10499.93	10519.67
10520.59	10550.21
10570.41	10609.67
10615.18	10661.56
10662.02	10706.79
10709.31	10759.59
10761.75	10793.93
10828.47	10867.50
10874.06	10913.30
10951.44	10975.30
11028.77	11084.03
11090.89	11123.96
11132.45	11172.40
11285.82	11324.39
11377.88	11401.99
11402.35	11426.50
11428.16	11457.32
11464.41	11490.90
11491.24	11511.70
11513.71	11548.26
11563.85	11591.40
11613.00	11640.84
11643.86	11670.36
11670.69	11702.22
11719.74	11745.69
11746.38	11765.89
11769.80	11802.40
11822.14	11848.77
11867.09	11899.68
11900.20	11926.61
11927.82	11948.69
11949.11	11980.79

11980.83	12027.80
12064.36	12096.27
12266.86	12313.47
12436.99	12481.07
12546.50	12600.69
12601.37	12629.15
12661.30	12688.16
12723.06	12753.59
12769.89	12808.24
12830.74	12911.09
12937.95	12988.01
13049.54	13104.18
13119.56	13151.02
13259.38	13288.08
13304.84	13347.09
13349.01	13382.93
13510.31	13538.16
13598.03	13641.19
13700.43	13739.69
13739.92	13823.03
13826.35	13864.02
13864.91	13924.11
13925.45	13959.08
13959.98	14002.58
14008.41	14076.57
14077.02	14122.31
14124.55	14179.70
14180.60	14228.58
14229.93	14279.70
14280.60	14323.20
14412.88	14457.73
14464.45	14514.23
14516.47	14570.28
14571.18	14618.26
14707.60	14746.88
14764.89	14808.84
14859.96	14904.35
14951.88	15008.38
15519.58	15601.20
15717.50	15784.83
16261.72	16344.23
16447.74	16561.02
16607.55	16644.36

17771.55	17847.33
17971.11	18021.03
18021.82	18074.32
18248.22	18311.89
18594.40	18682.74
18811.43	18885.87
19822.65	20176.24
20808.15	20854.43
20873.86	21004.45
21006.87	21124.88
21125.85	21221.13
21221.61	21322.70
21323.18	21423.30
21636.47	21936.48
22991.54	23107.22
23107.99	23203.63
23205.94	23327.80
23404.15	23535.26
27895.09	27996.12
27999.21	28148.83

Biological processes

The following table lists the investigated biological processes, and the associated GO terms in the database search. The last column, N of proteins, lists the number of proteins that are associated with a particular proteins and is measured in the SOMAscan panel.

**Biological Process**	**Searched GO processes**	**N of proteins**
Acute inflammatory response	GO:0002541; GO:0002526; GO:0002438; GO:0002674; GO:0002675; GO:0002673	13
Acute phase reaction	GO:0006953	14
Complement activation	GO:0006956; GO:0006957; GO:0006958; GO:0001867; GO:0045916; GO:0045957; GO:0045959; GO:0001869; GO:0045917; GO:0030449	46
Cytokine production involved in immune response	GO:0002368; GO:0035743; GO:0002367; GO:0002374; GO:0002371; GO:0010934; GO:0032762; GO:1900016; GO:0002740; GO:0002731; GO:0010936; GO:0002737; GO:0002725; GO:2000552; GO:0002720; GO:1900017; GO:0002741; GO:0060907; GO:0032765; GO:0002729; GO:0002726; GO:2000556; GO:2000553; GO:0002721; GO:0002718;	31

	GO:1900015; GO:0002724; GO:0002369; GO:0035745	
Response to hypoxia	GO:0071456; GO:0070483; GO:0097411; GO:1990144; GO:1900038; GO:1903298; GO:0061428; GO:1902073; GO:2000777; GO:0061419; GO:1900037; GO:0061418; GO:0001666	41
Extracellular matrix organization	GO:0070831; GO:0034769; GO:0071711; GO:0030199; GO:0048251; GO:0085029; GO:0070278; GO:0022617; GO:0030198; GO:0035426; GO:1905590; GO:0032836; GO:1904027; GO:0010716; GO:1903054; GO:1904261; GO:1904028; GO:1901203; GO:0003331; GO:0090091; GO:1901201; GO:0010715	97
Epithelial-mesenchymal transition	GO:0044334; GO:0001837; GO:0060809; GO:0010719; GO:0010718; GO:0010717	20
Angiogenesis	GO:0001525; GO:0060978; GO:0060055; GO:0002043; GO:0001569; GO:0120078; GO:0060981; GO:0002042; GO:0097102; GO:0072277; GO:0016525; GO:1903588; GO:0090051; GO:1903671; GO:1905555; GO:0045766; GO:1903589; GO:0090050; GO:1903672; GO:0035470; GO:0045765; GO:1903670; GO:0061043; GO:0002040; GO:0061042	109
IFN type 1 signalling/ response	GO:0071357; GO:0032607; GO:0032608; GO:0045355; GO:0032687; GO:0032688; GO:0035548; GO:0032480; GO:0060339; GO:0045356; GO:0032727; GO:1902741; GO:0032728; GO:0032481; GO:0060340; GO:0032648; GO:0032479; GO:0060338; GO:0034340; GO:0060337	31
IFN γ signalling/ response	GO:0071346; GO:0060333; GO:0045078; GO:0032729; GO:1902715; GO:0060335; GO:0032649; GO:0060334; GO:0034341; GO:0042095; GO:0032609; GO:0004906; GO:0072643	68
Glycolysis	GO:0061621; GO:0006096; GO:0061615; GO:0045821; GO:0006110	20
Behavior	GO:0007631; GO:0007626; GO:0007611; GO:0007613; GO:0007612; GO:0007616; GO:0003051; GO:0007632; GO:0008344; GO:0042755; GO:0035640	21
Cellular component morphogenesis	GO:0032989; GO:0010927; GO:0032990;GO:0000902	44
Immune tolerance and suppression	GO:0002361; GO:0002519; GO:0032834; GO:0045591; GO:0002458; GO:0042092; GO:0045589; GO:0035745; GO:2000553; GO:0045630; GO:0045066; GO:0045590; GO:0002859; GO:2000521; GO:2001189; GO:0002830; GO:0045626; GO:0002826; GO:0045064; GO:0002362; GO:0032831; GO:0002845; GO:0002513; GO:0046007; GO:0060336; GO:0050727; GO:0050729	21
Chronic inflammatory response	GO:0002544; GO:0002439; GO:0002678; GO:0002876; GO:0002882; GO:0002676	9
Type 17 immune response	GO:0072540; GO:2000318; GO:0072539; GO:0072540; GO:2000318; GO:0072539; GO:2000318; GO:0072540; GO:0072539; GO:2000318; GO:2000318; GO:2000321; GO:0072540; GO:2000321; GO:2000321; GO:0072540; GO:2000318; GO:2000321; GO:0072538	13
Innate immune response	GO:0002218; GO:0045087; GO:0045089; GO:0045088	72
Wound healing	GO:2001114; GO:0035195; GO:0051683; GO:0090168; GO:2001028; GO:0042060; GO:0045893; GO:0060070; GO:0060071; GO:0061041;	49

	GO:0061042; GO:0061045; GO:0090303; GO:0044319; ; GO:0061044; GO:0035470; GO:0035313; GO:0002246; GO:1903691; GO:1903690; GO:0060054; GO:0002248; GO:0044330; GO:0042246; GO:0001778; GO:0007596; GO:0030168; GO:0043403; GO:1905041; GO:0030194; GO:0010572; GO:0002543; GO:0030195; GO:0051919; GO:0042730; GO:0051917; GO:0030193; GO:0010543; GO:0070527; GO:0014834; GO:0051918; GO:0090330; GO:0072378; GO:0007597; GO:0072377; GO:0010544; GO:0090331; GO:1901731; GO:0097045	
