# Supplementary figures and images for: Extending the information content of the MALDI analysis of biological fluids via multi-million shot analysis
Source: PLoS One. 2019 Dec 9;14(12):e0226012. doi: 10.1371/journal.pone.0226012 (PMC6901224; doi:10.1371/journal.pone.0226012)

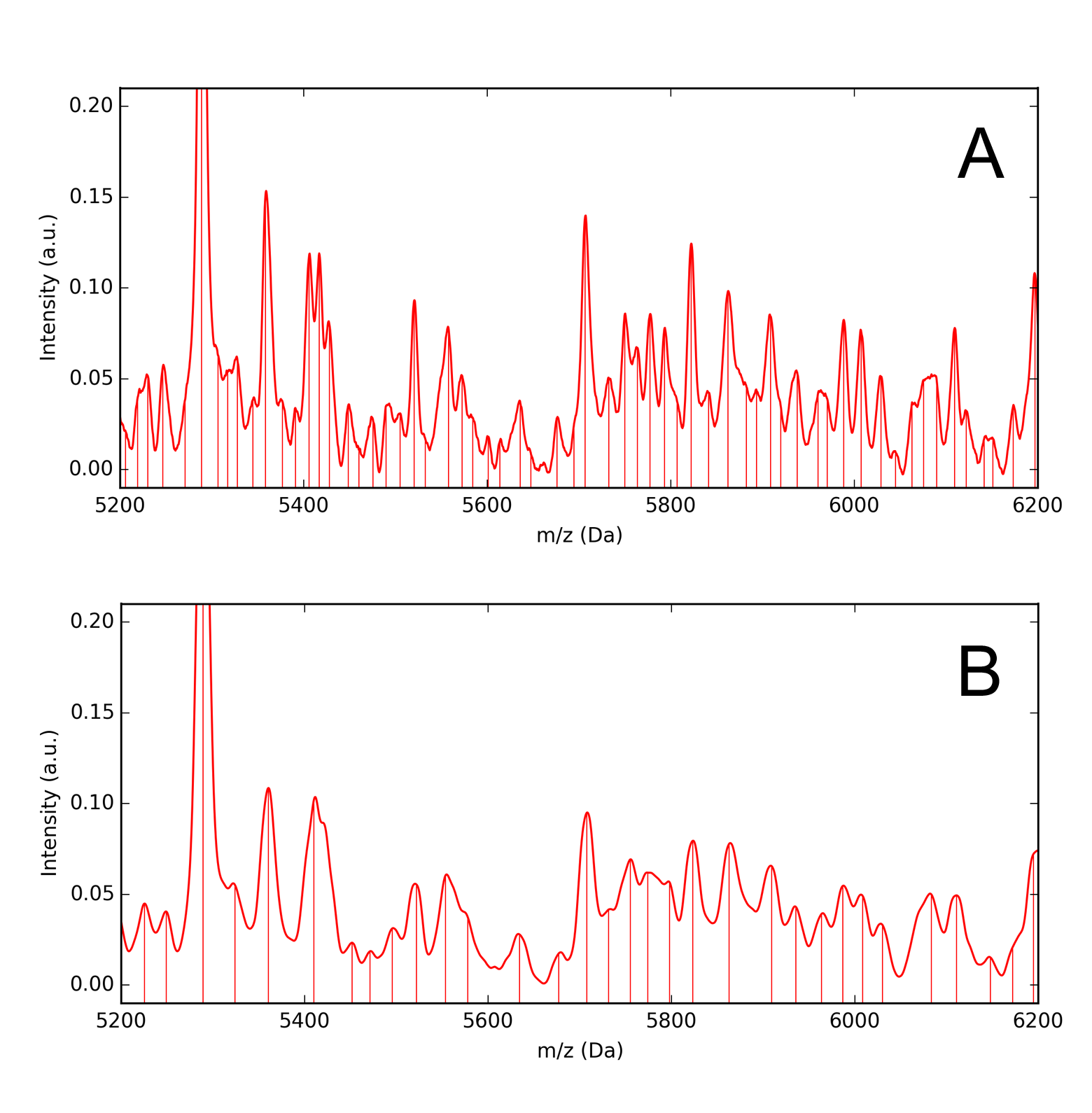

Supplement: S1 Fig — A) Average spectrum acquired on SimulTOF100, 100 million laser shots. 62 peaks are detected in the m/z range shown. B) The same spectrum with resolution artificially reduced by a factor of 2 by applying a moving average filter. The number of detected peaks is decreased to 32. The signal/noise ratio threshold for peak detection (SNR = 10) is the same in both A and B. (TIF) [file pone.0226012.s002.tif]

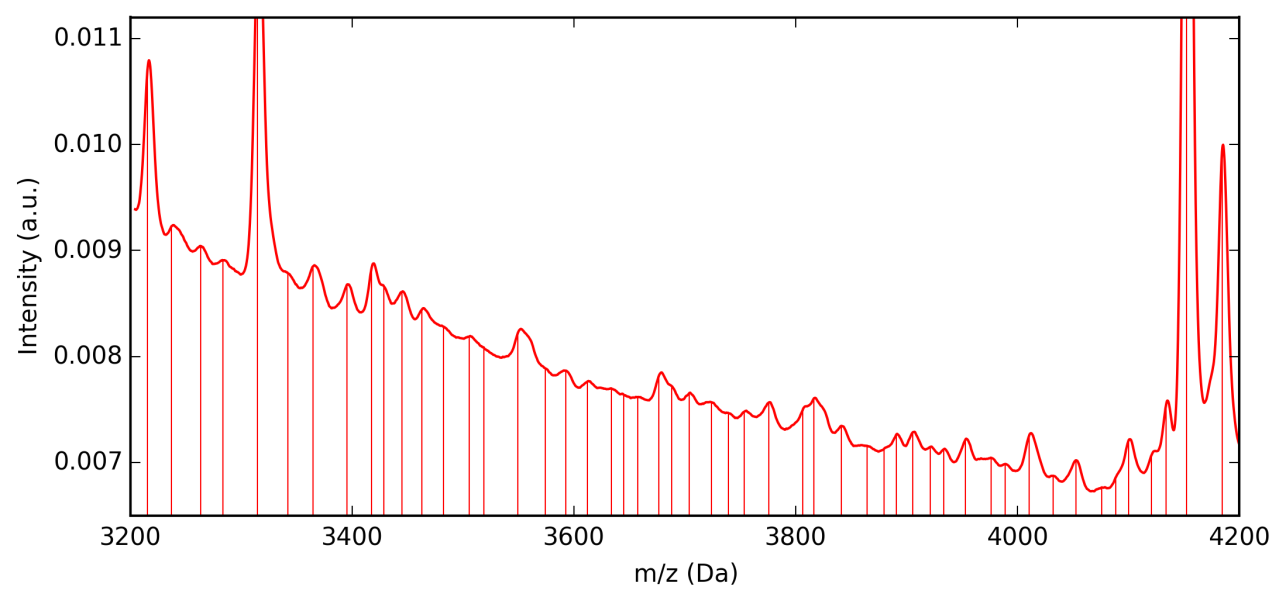

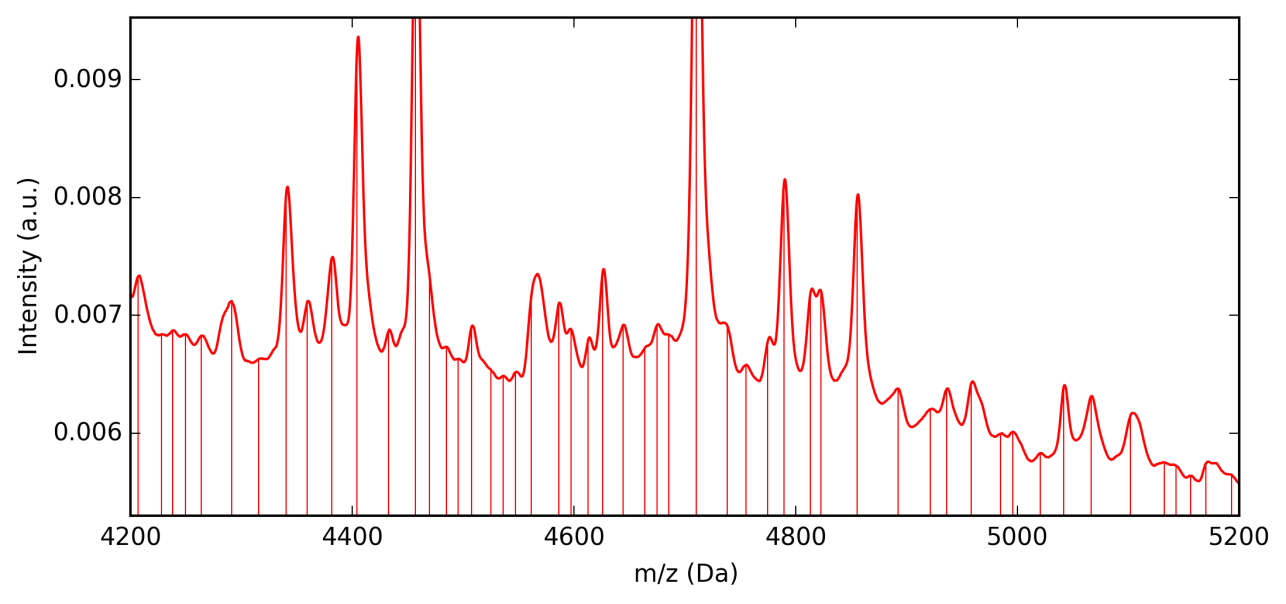

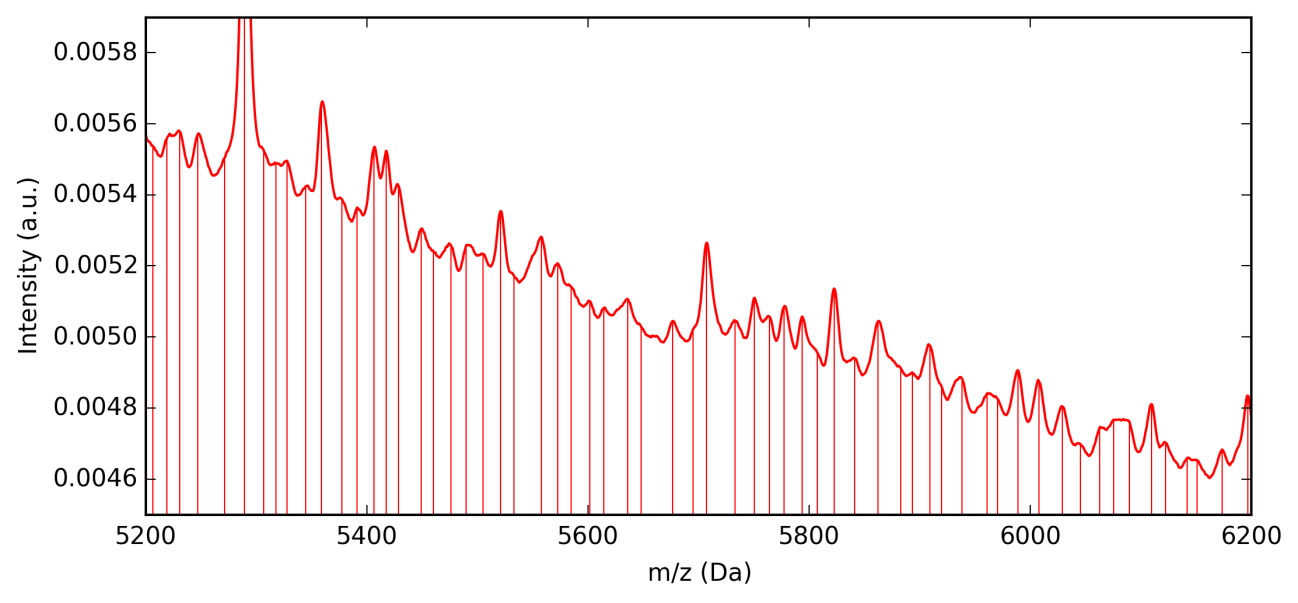

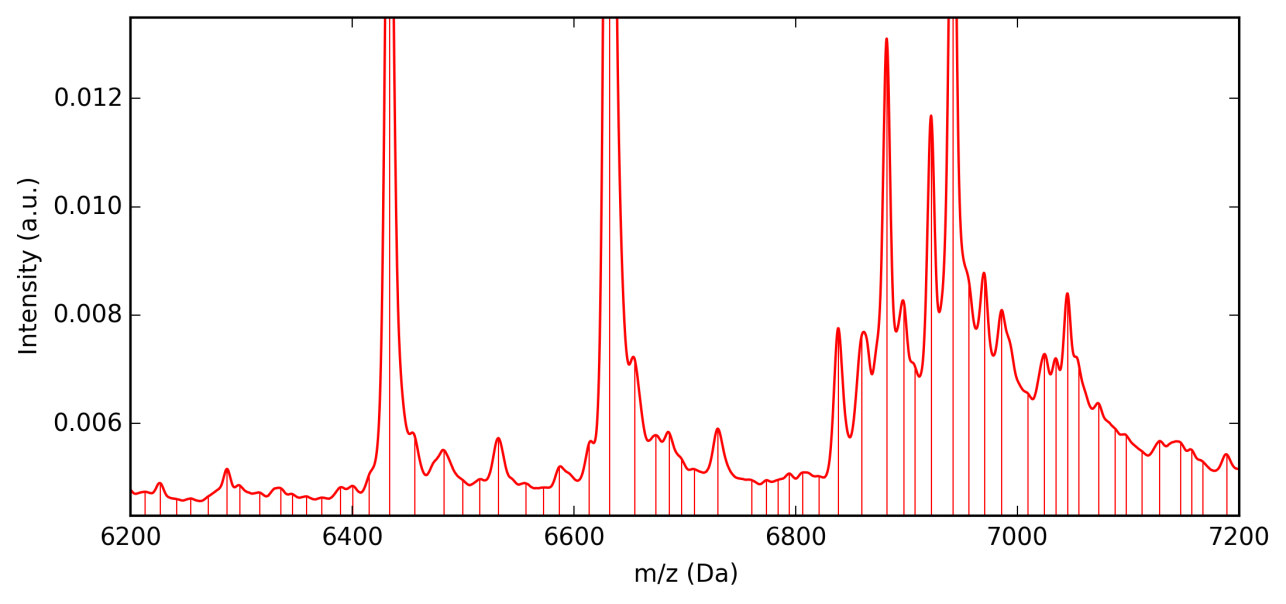

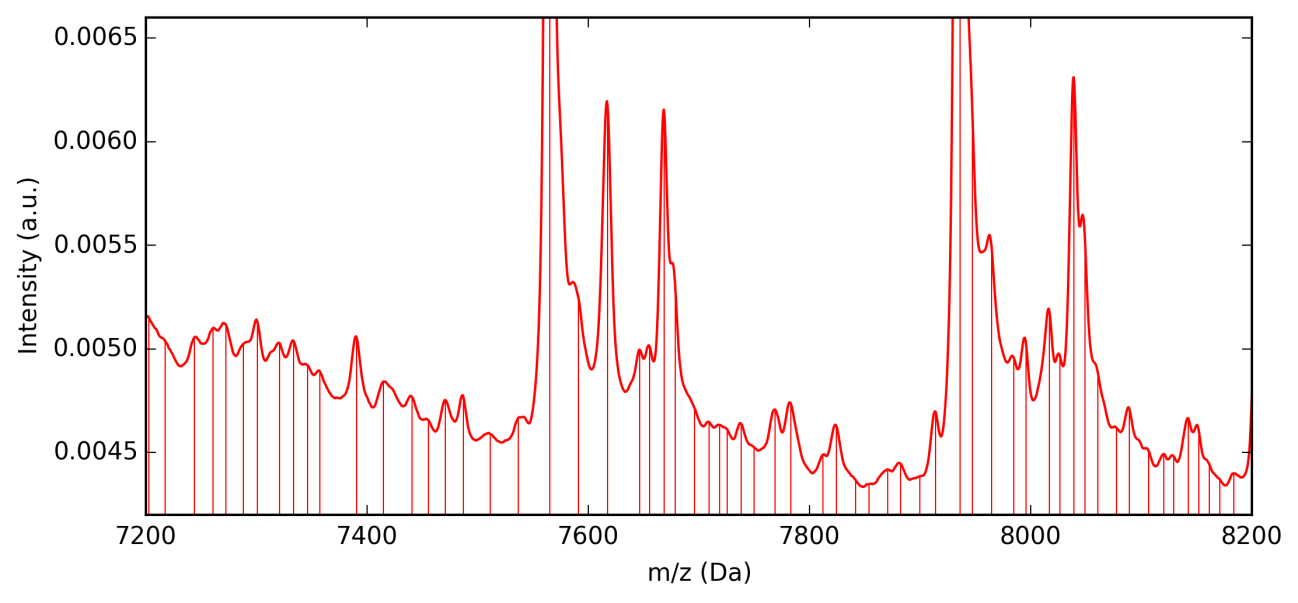

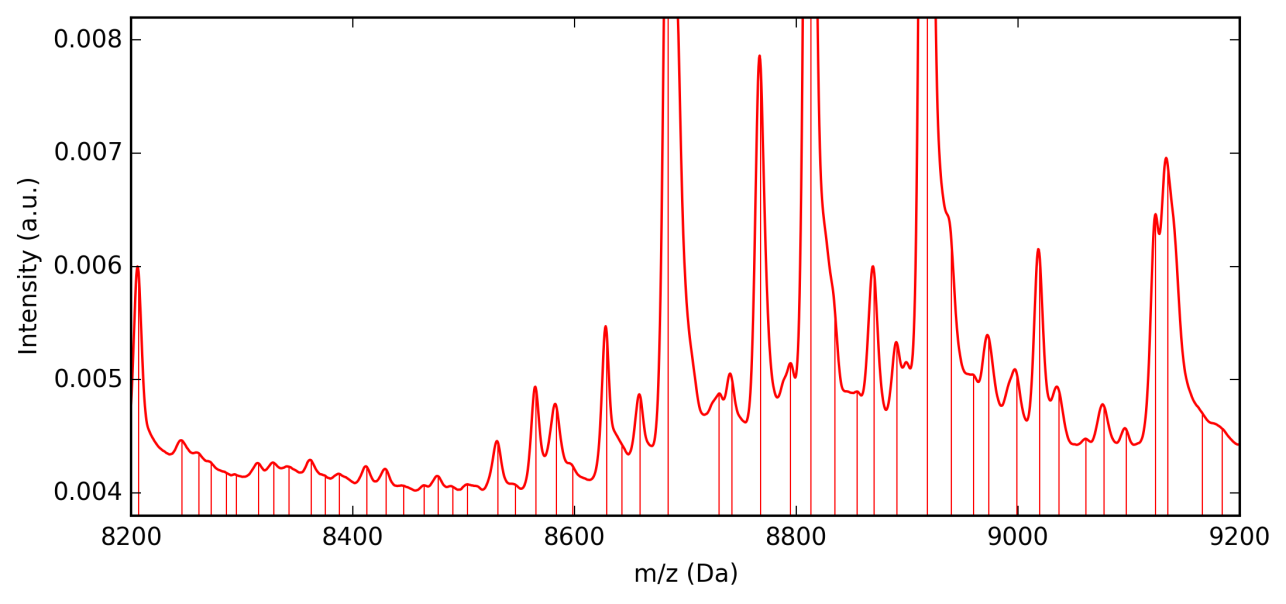

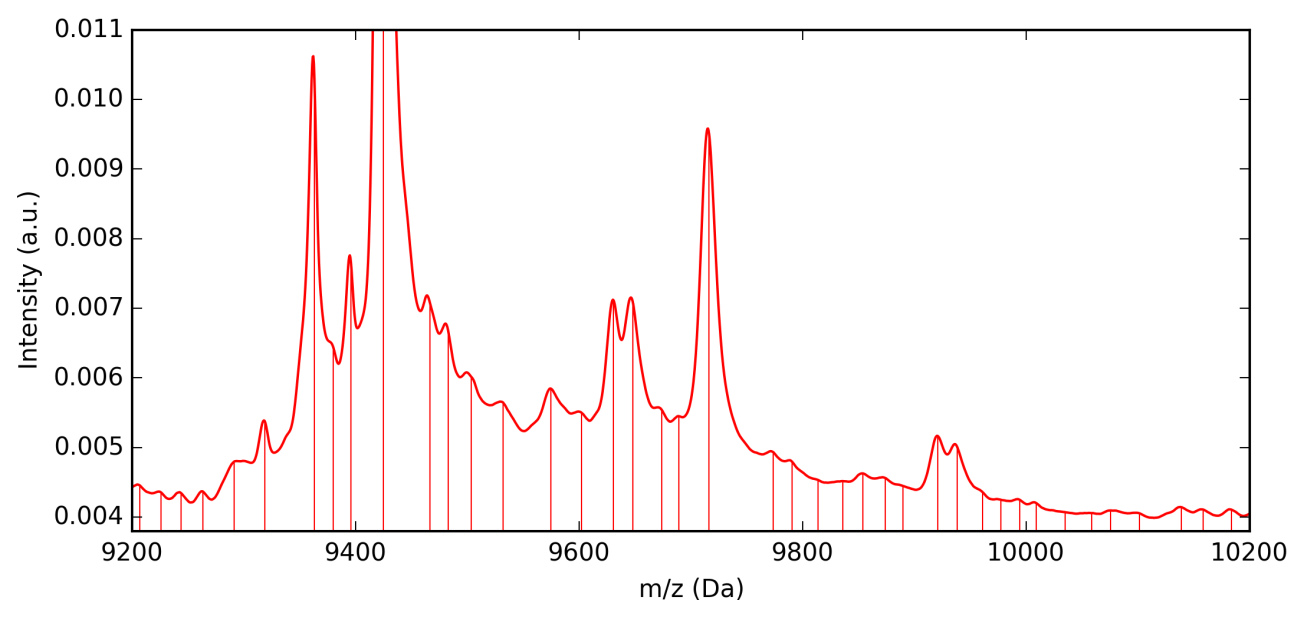

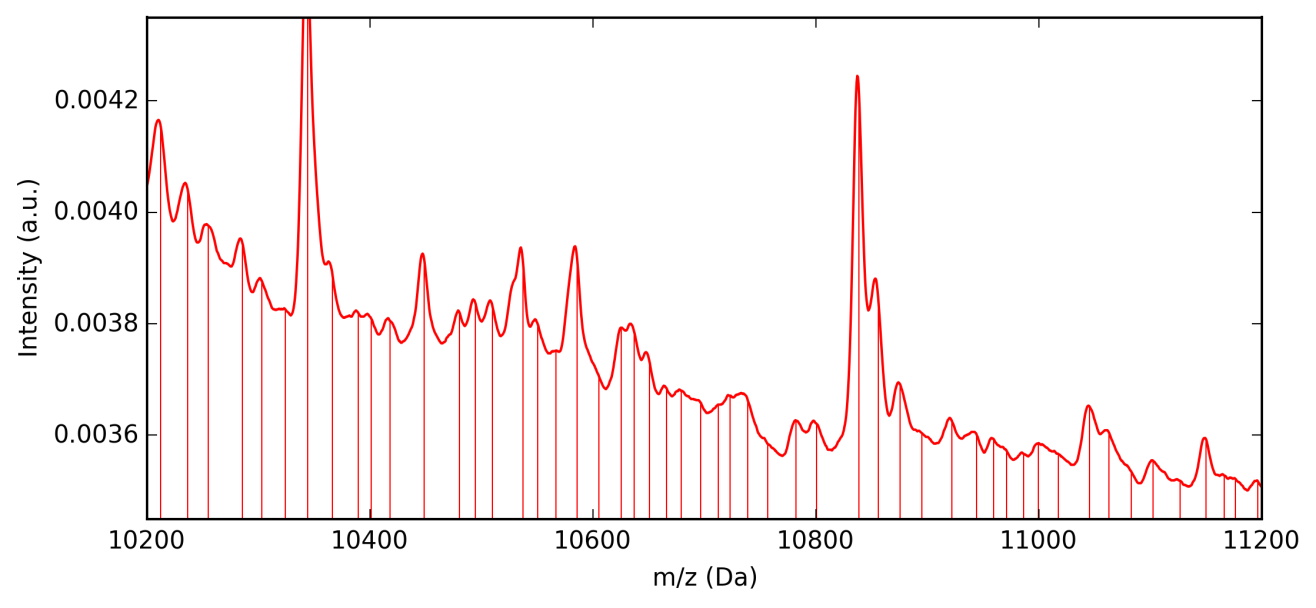

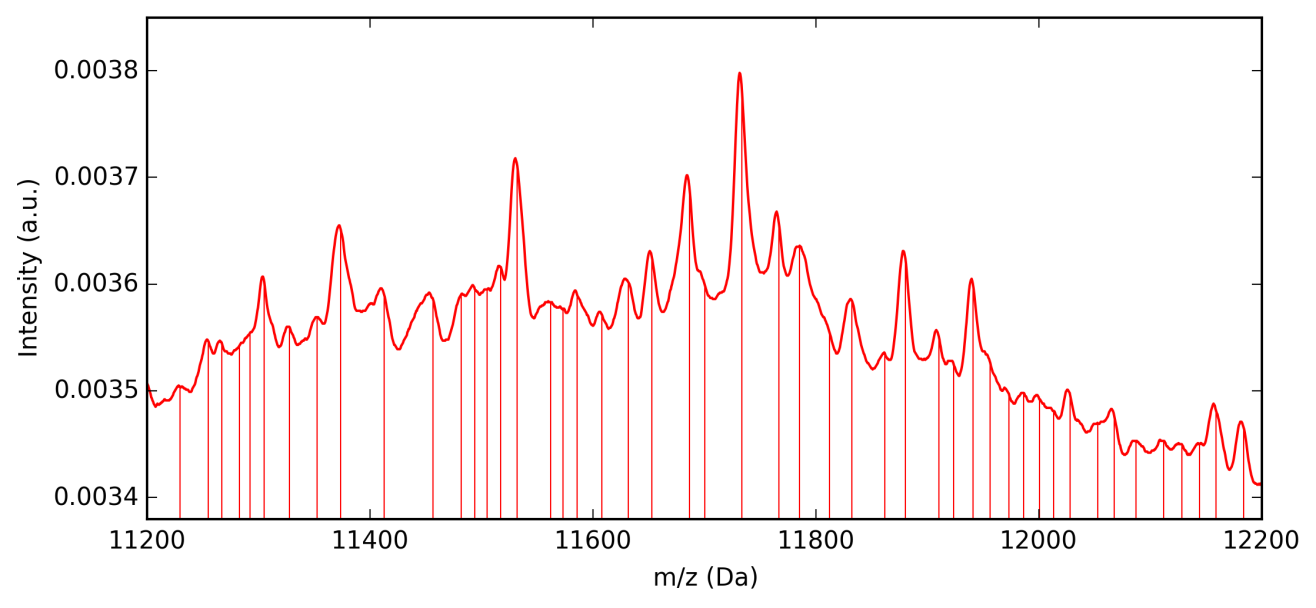

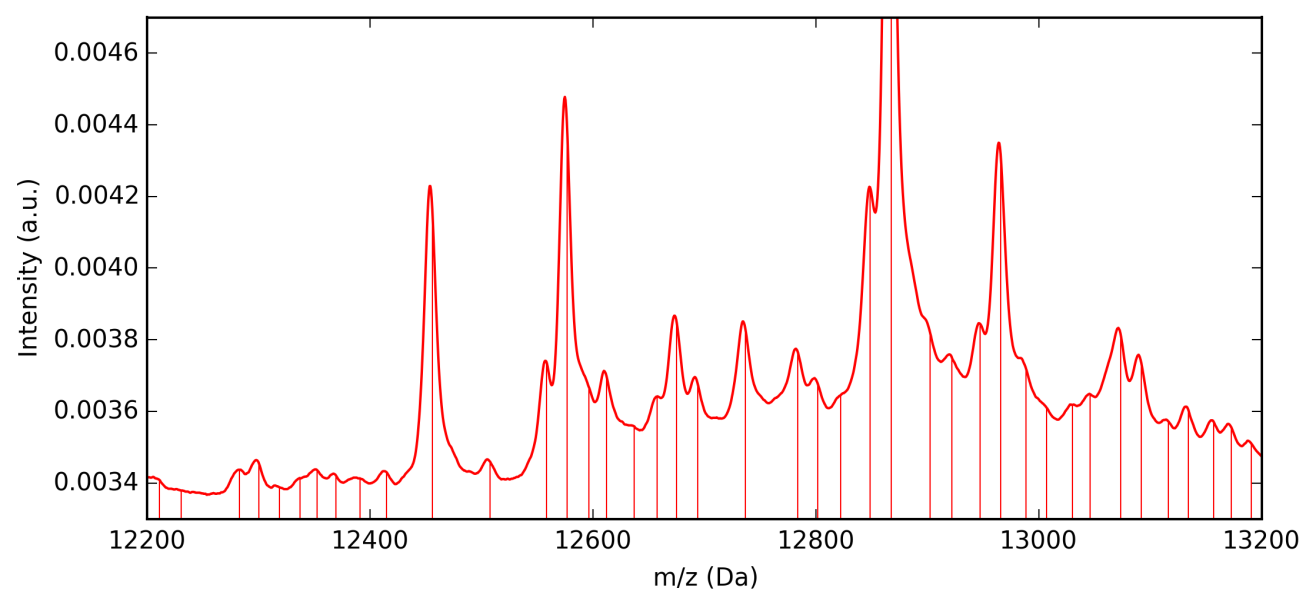

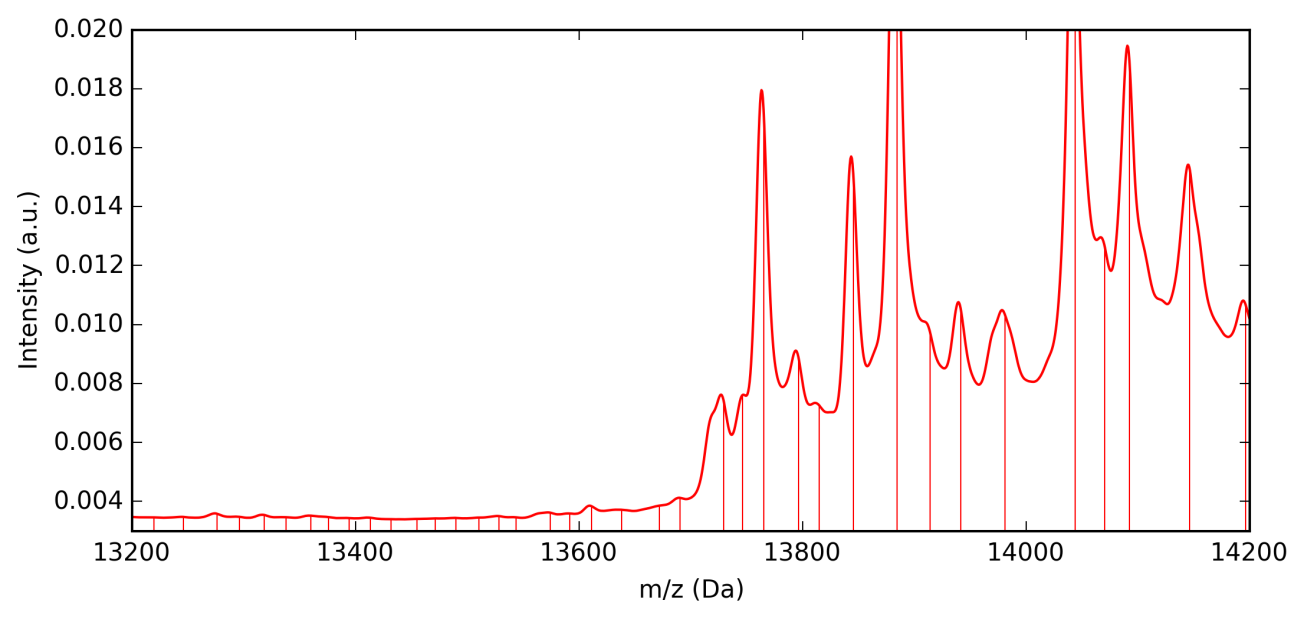

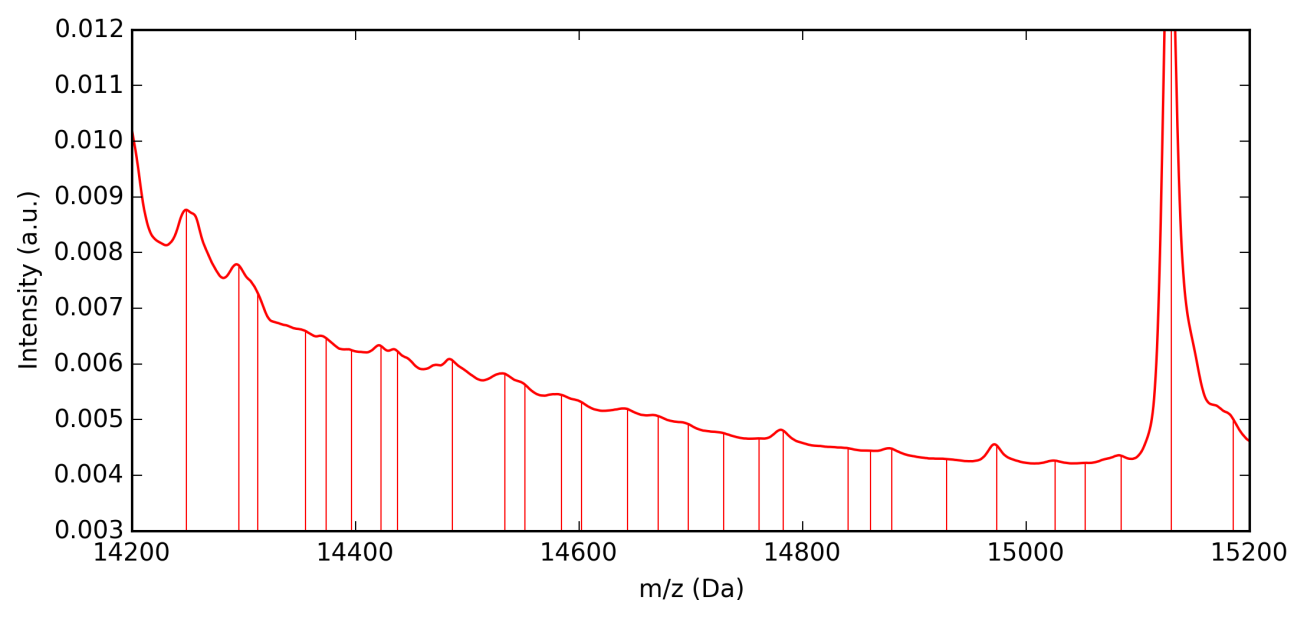

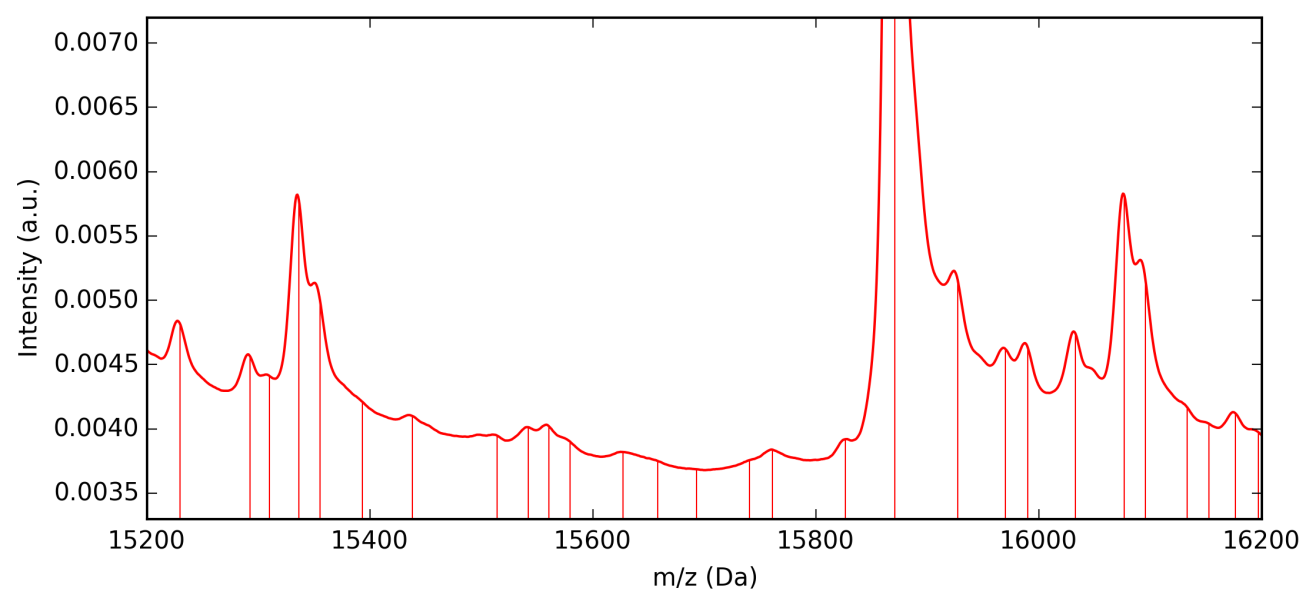

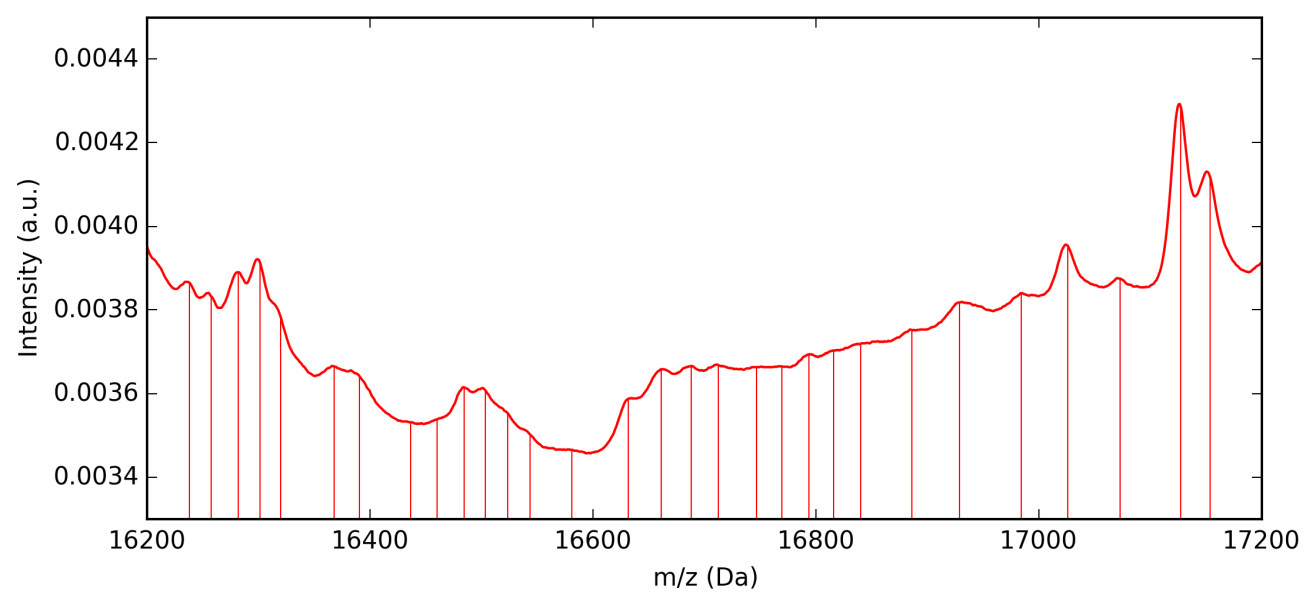

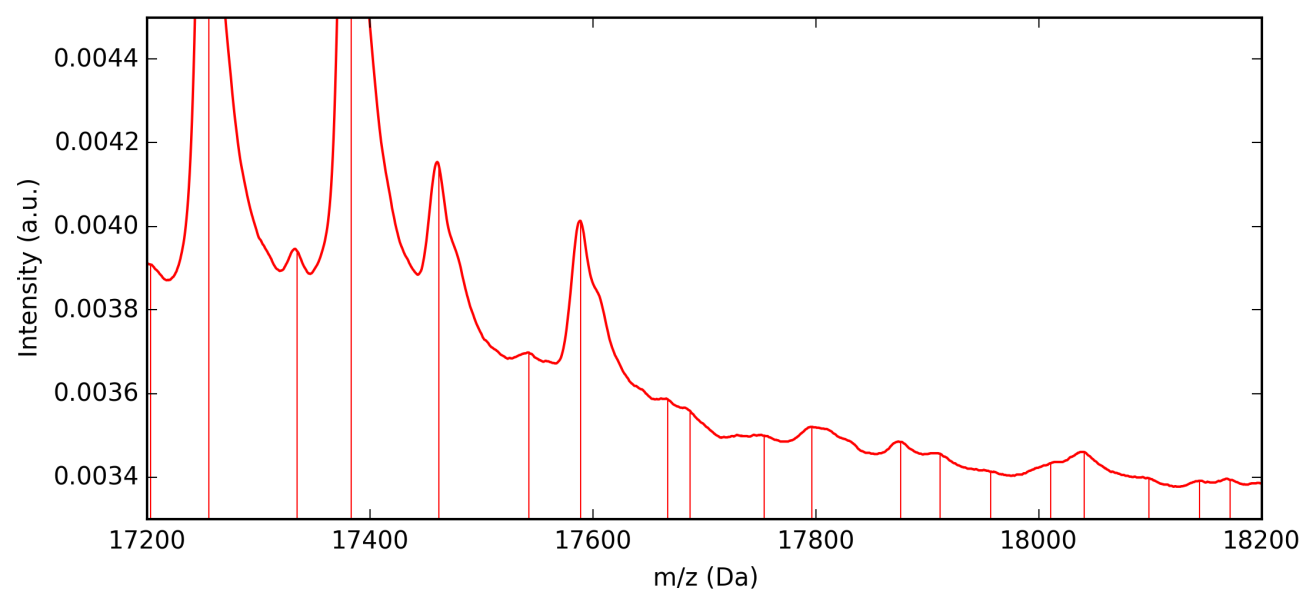

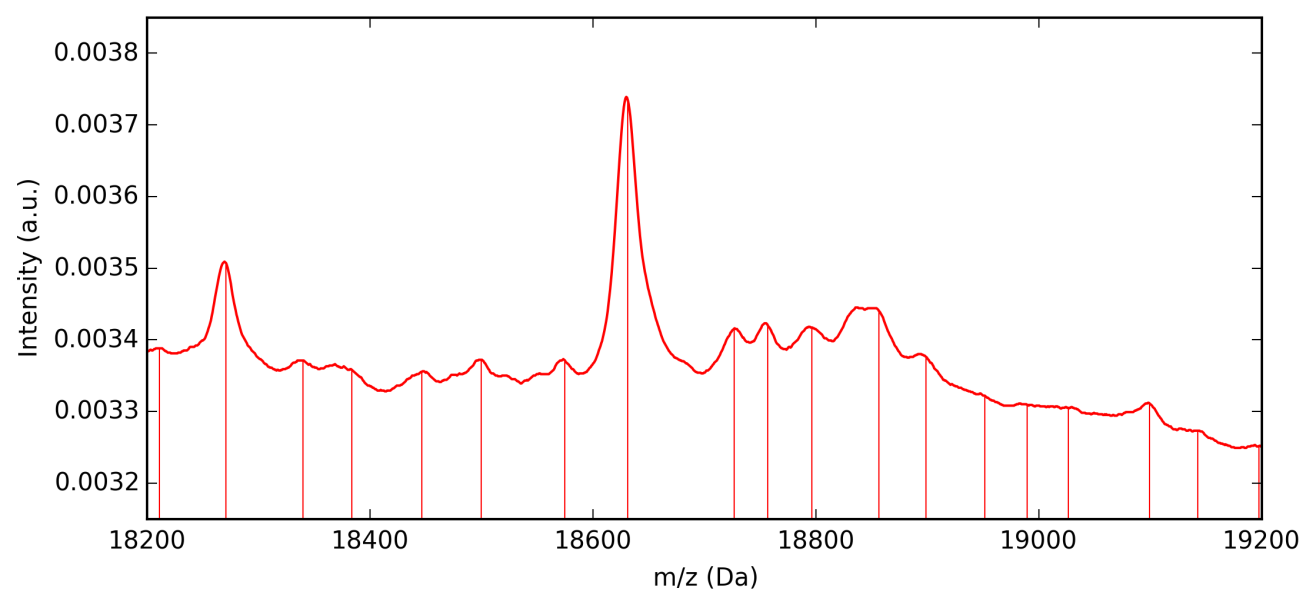

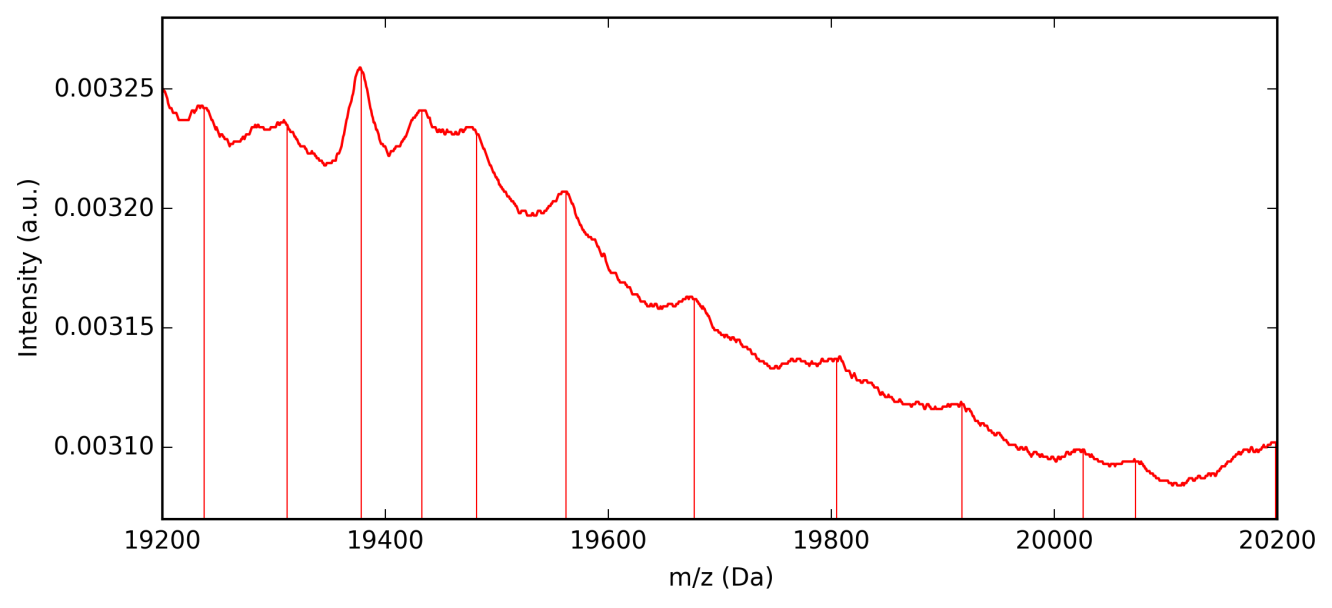

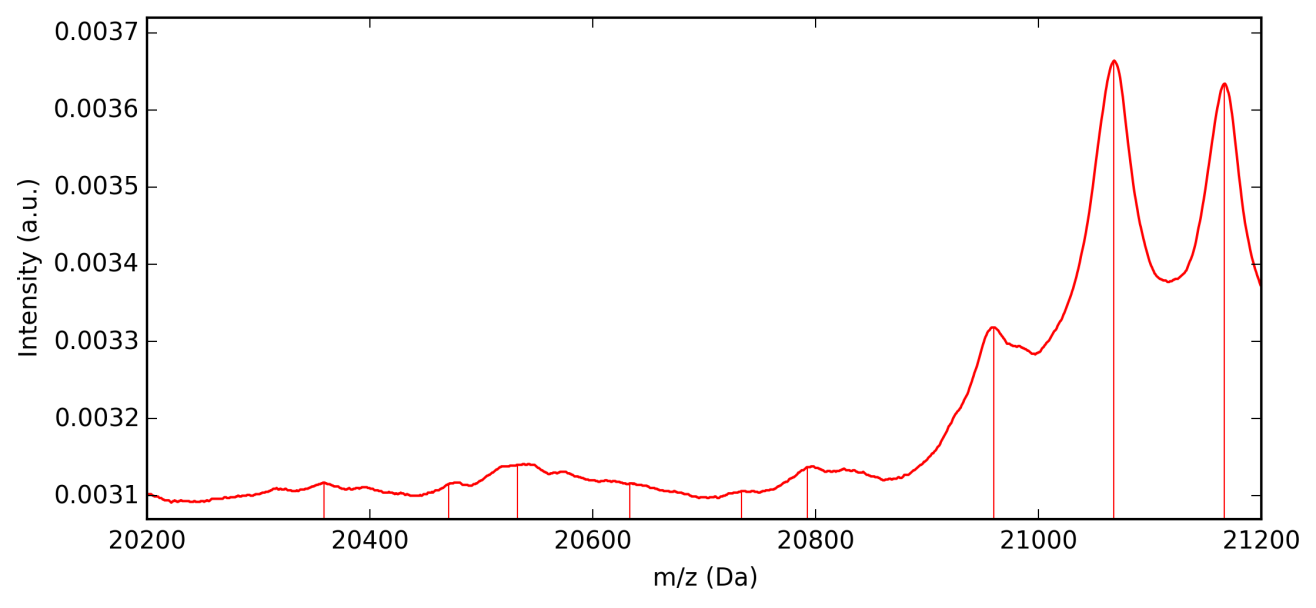

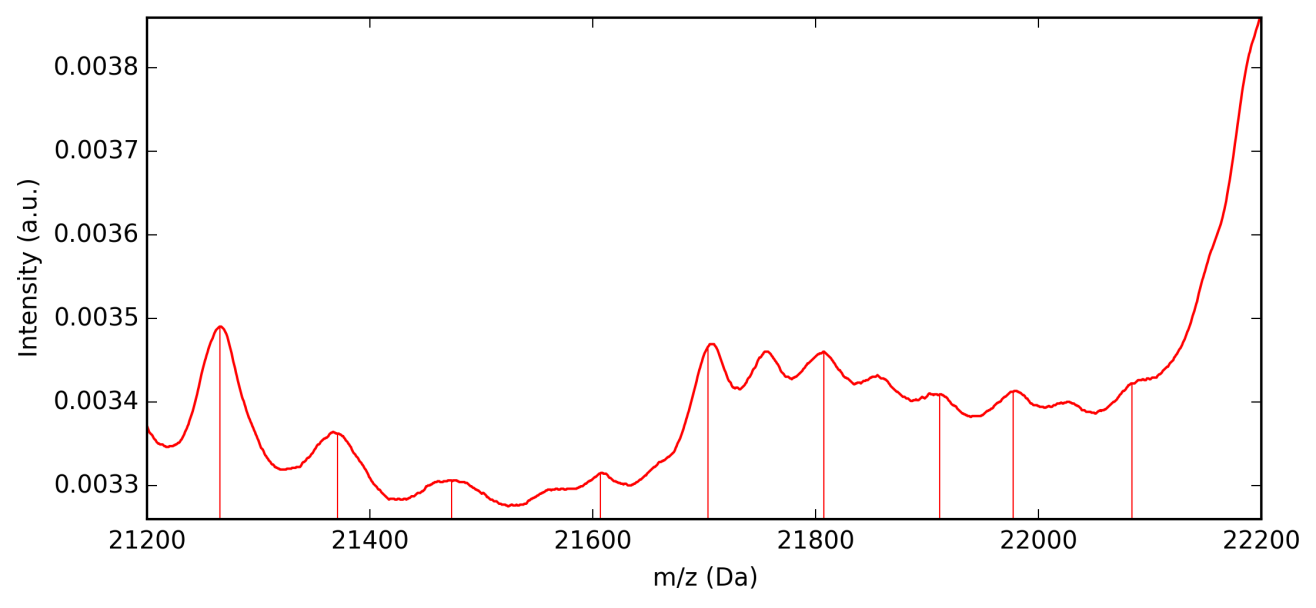

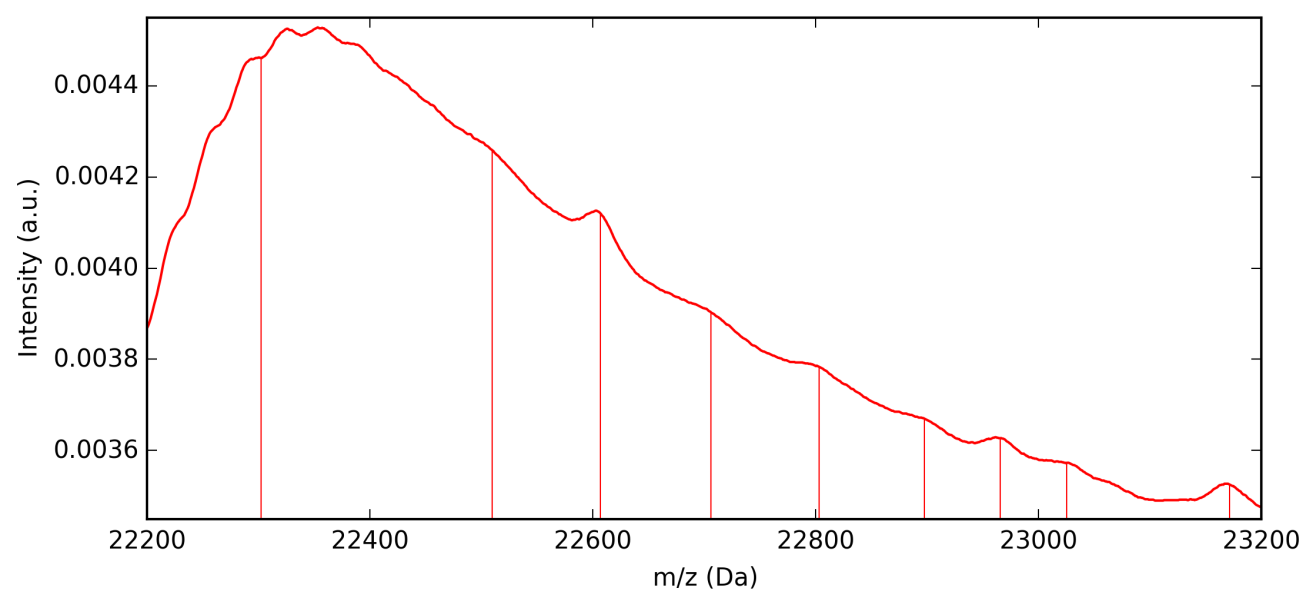

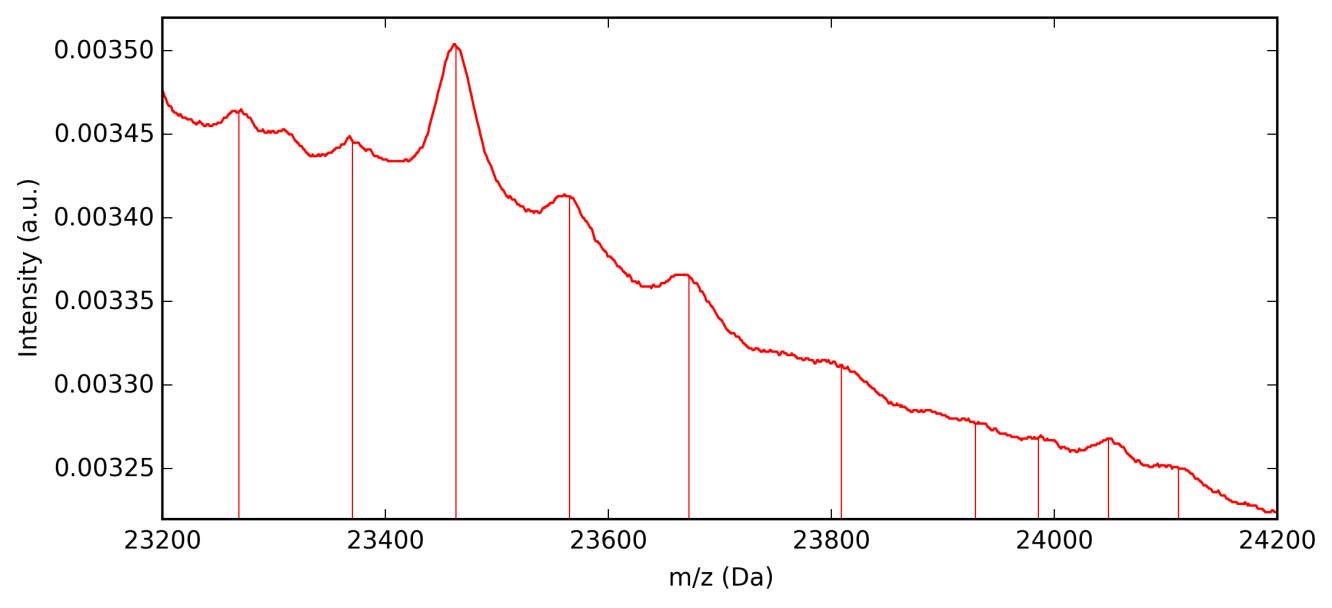

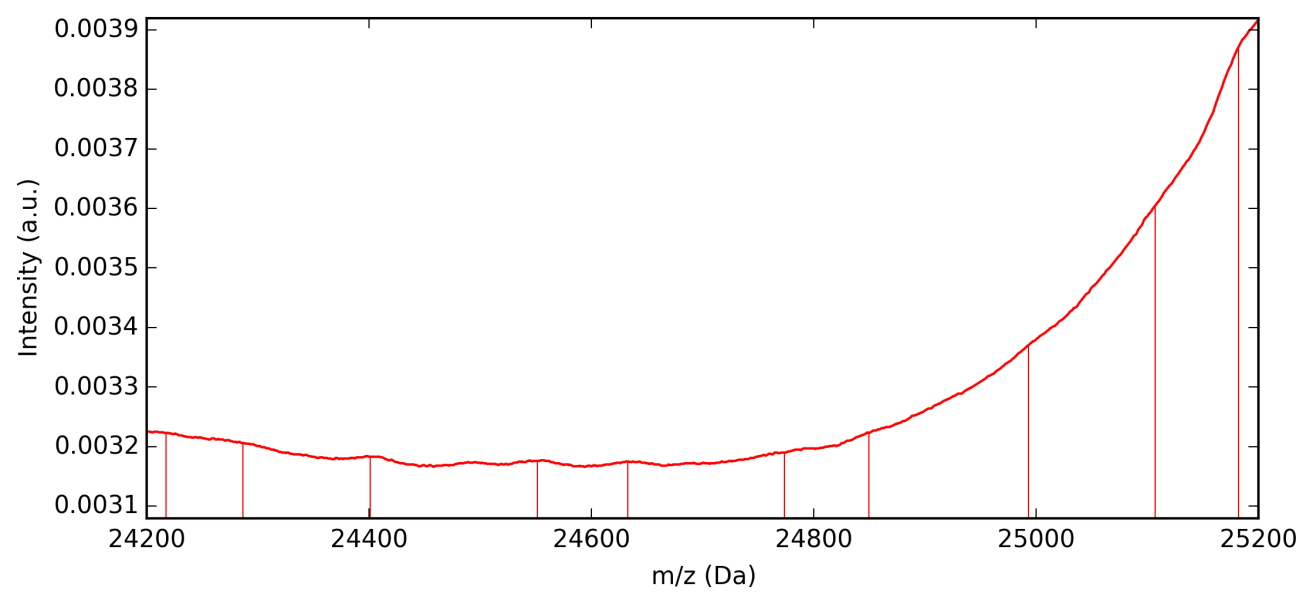

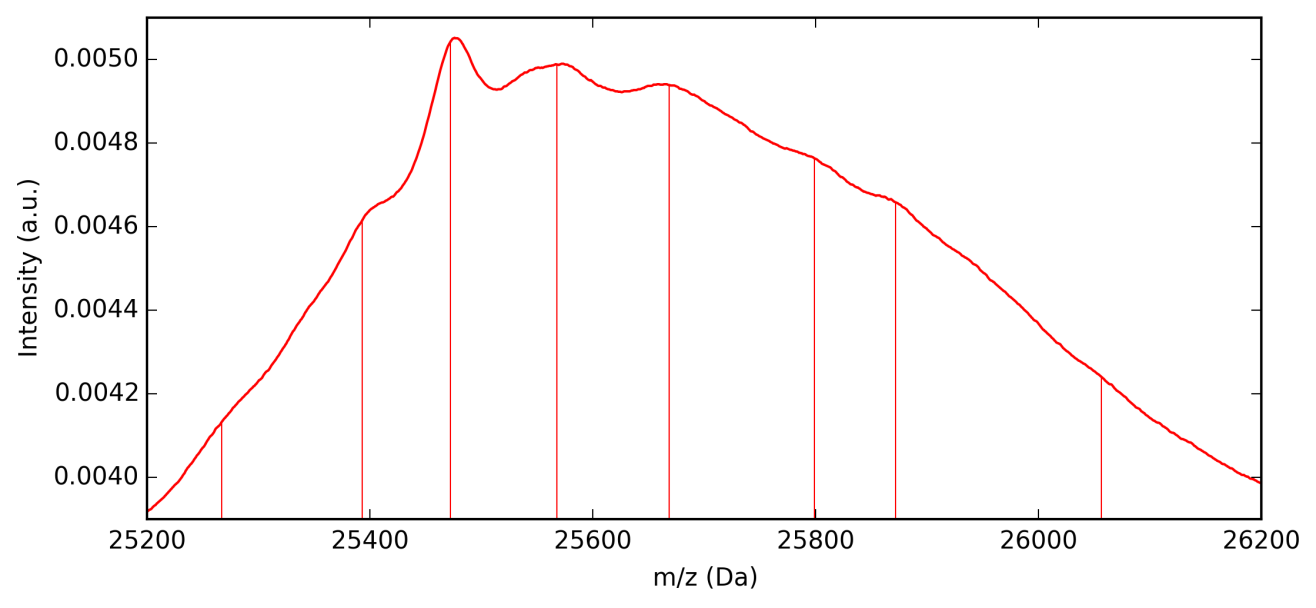

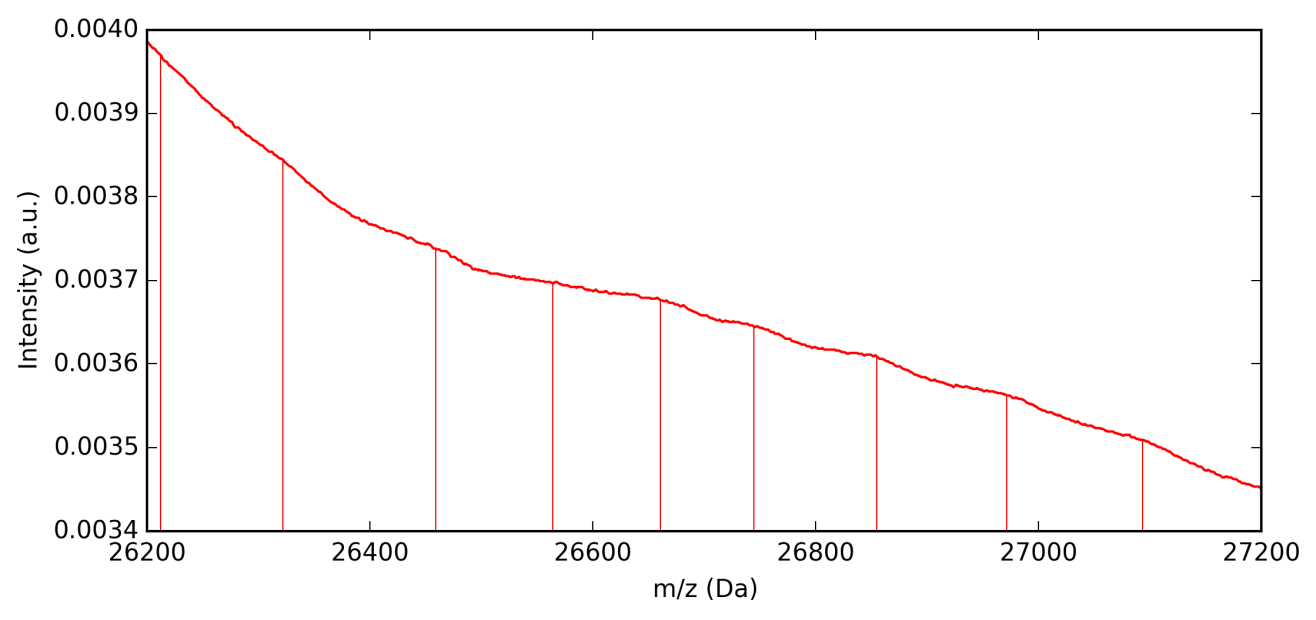

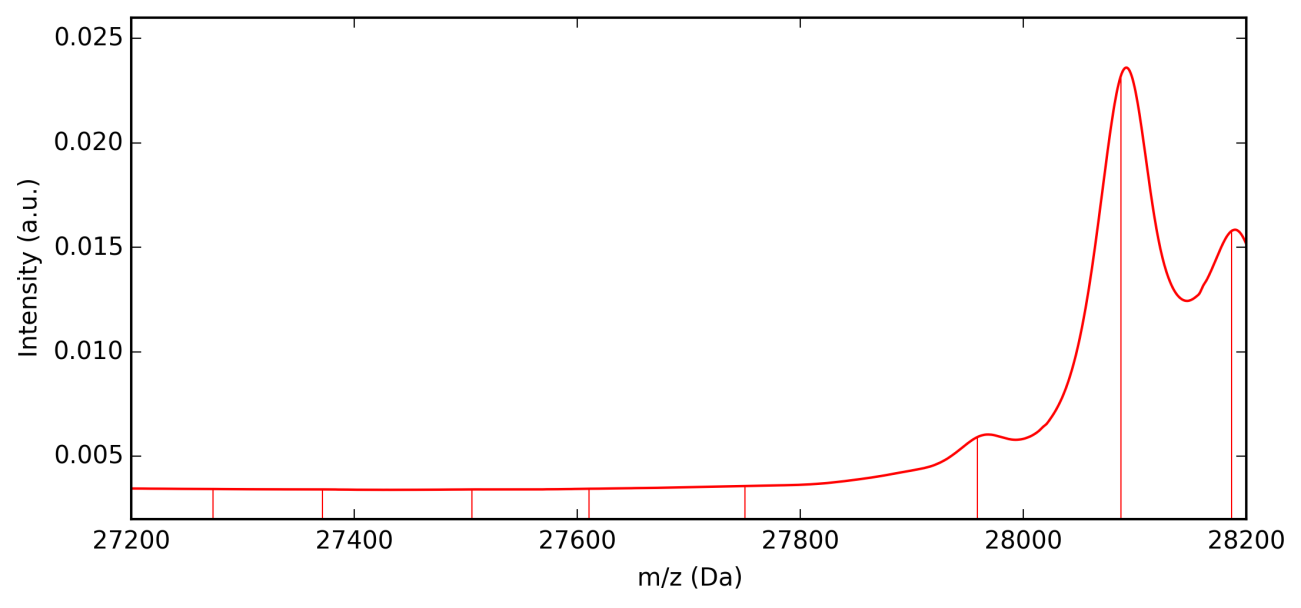

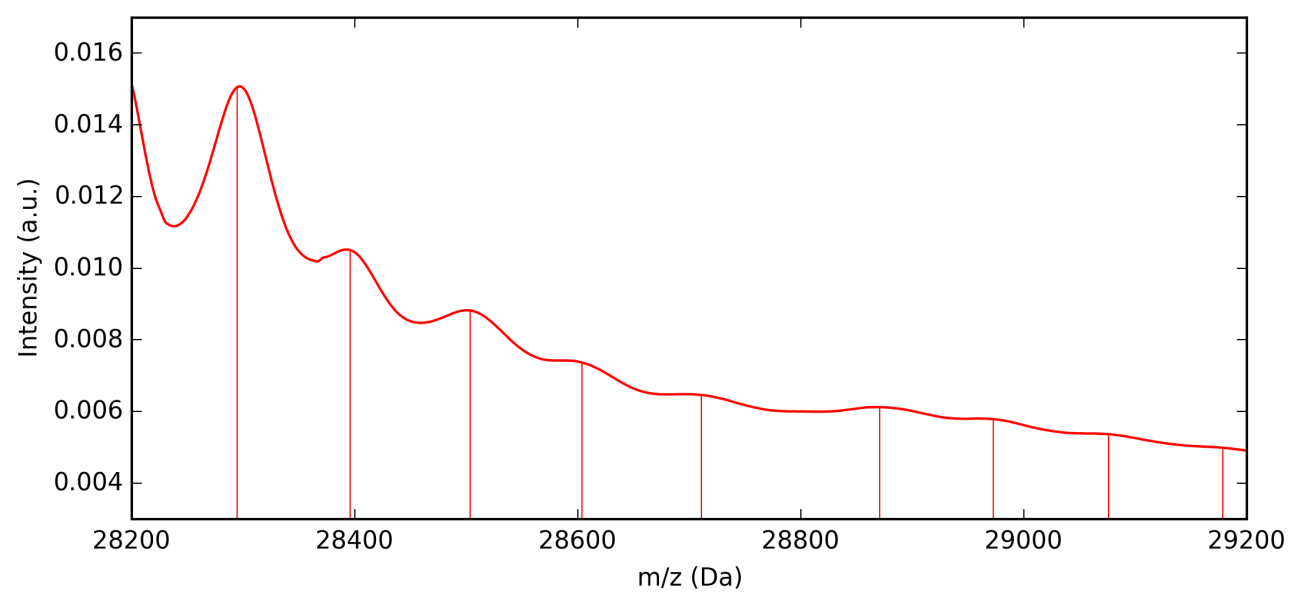

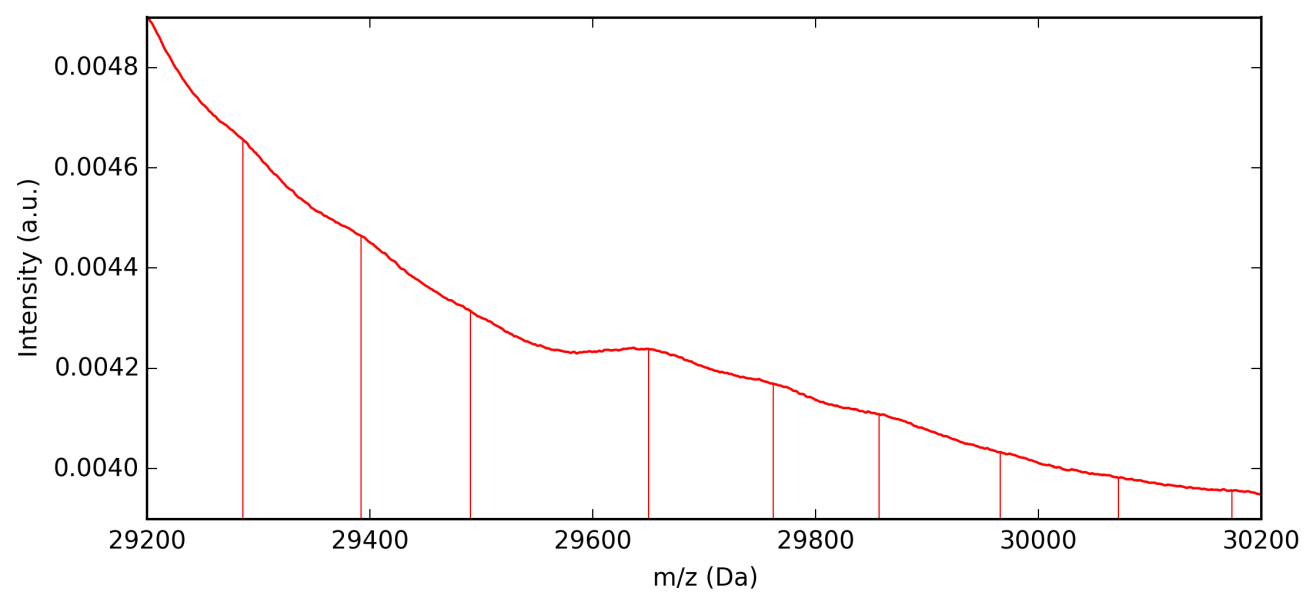

Supplement: S2 Fig — The 100 million shot spectrum is plotted as a function of m/z. Detected peaks are marked by vertical lines. (DOCX) [file pone.0226012.s003.docx]
